# Pre-marked chromatin and transcription factor co-binding shape the pioneering activity of Foxa2

**DOI:** 10.1093/nar/gkz627

**Published:** 2019-07-27

**Authors:** Filippo M Cernilogar, Stefan Hasenöder, Zeyang Wang, Katharina Scheibner, Ingo Burtscher, Michael Sterr, Pawel Smialowski, Sophia Groh, Ida M Evenroed, Gregor D Gilfillan, Heiko Lickert, Gunnar Schotta

**Affiliations:** 1 Division of Molecular Biology, Biomedical Center, Faculty of Medicine, LMU Munich, Germany; 2 Helmholtz Zentrum München, Institute of Stem Cell Research, Neuherberg, Germany; 3 Helmholtz Zentrum München, Institute of Diabetes and Regeneration Research, Neuherberg, Germany; 4 Bioinformatic Core Facility, Biomedical Center, LMU Munich, Martinsried, Germany; 5 Department of Medical Genetics, Oslo University Hospital and University of Oslo, Oslo, Norway; 6 German Center for Diabetes Research (DZD), Neuherberg, Germany; 7 Technische Universität München, Germany; 8 Munich Center for Integrated Protein Science (CiPSM), Munich, Germany

## Abstract

Pioneer transcription factors (PTF) can recognize their binding sites on nucleosomal DNA and trigger chromatin opening for recruitment of other non-pioneer transcription factors. However, critical properties of PTFs are still poorly understood, such as how these transcription factors selectively recognize cell type-specific binding sites and under which conditions they can initiate chromatin remodelling. Here we show that early endoderm binding sites of the paradigm PTF Foxa2 are epigenetically primed by low levels of active chromatin modifications in embryonic stem cells (ESC). Priming of these binding sites is supported by preferential recruitment of Foxa2 to endoderm binding sites compared to lineage-inappropriate binding sites, when ectopically expressed in ESCs. We further show that binding of Foxa2 is required for chromatin opening during endoderm differentiation. However, increased chromatin accessibility was only detected on binding sites which are synergistically bound with other endoderm transcription factors. Thus, our data suggest that binding site selection of PTFs is directed by the chromatin environment and that chromatin opening requires collaboration of PTFs with additional transcription factors.

## INTRODUCTION

Transcription factors (TFs) drive lineage-specific transcription programs by binding gene regulatory elements dispersed throughout the genome (1). However, since DNA is wrapped around histones to form nucleosomes and chromatin, TFs have to overcome this physical barrier to bind their DNA target sites (2,3). Although most TFs can recognize their target sequence only on nucleosome-free DNA, so-called pioneer transcription factors (PTFs) have the peculiar ability to engage their target sequence on nucleosomal DNA (4,5). Following binding to their target sites, PTFs can induce chromatin opening supporting the recruitment of non-pioneer TFs and ultimately leading to activation of the underlying gene regulatory elements (6,7). Interestingly, despite their potentially universal targeting, PTFs only bind to a subset of their potential DNA binding motif containing target sites (6,8–9). These findings imply that additional mechanisms, such as cell-type specific cofactors (10,11) and chromatin environment (12–15) can influence binding site selection of PTFs.

While it is widely recognized that PTFs have the capacity to engage with previously inaccessible regions of chromatin, there is still scarce understanding of how they initiate remodelling and opening of the surrounding chromatin. Binding of PTFs can lead to eviction of nucleosomes (16) or displacement of linker histone H1 (17). However, it is currently unclear how PTFs assemble distinct chromatin remodelling machineries on specific binding sites.

We have tackled those questions by studying the paradigm PTF Foxa2 in the physiological context of *in vitro* endoderm differentiation from mouse ESCs. We found that Foxa2 binding during endoderm differentiation is dynamic with stable and differentiation stage-specific binding sites. Endoderm-specific Foxa2 binding sites feature low levels of active chromatin modifications in ESCs, suggesting an epigenetic priming for Foxa2 recruitment during differentiation. We found that Foxa2 binding is required but not sufficient for chromatin opening. Rather, co-binding of Foxa2 with additional endoderm TFs appears necessary for chromatin opening. In summary, our data suggest that binding sites for pioneer transcription factors are epigenetically primed and that chromatin opening requires synergistic binding of transcription factors in close vicinity.

## MATERIALS AND METHODS

### Endoderm differentiation of DKI mESCs

DKI ESCs (Foxa2-Venus heterozygous; Sox17-Cherry homozygous) (18,19) were thawed on gamma-irradiated feeders and maintained undifferentiated in ESC medium based on DMEM (12634028, Gibco) containing 15% FCS, mLIF (self-made), 12 ml HEPES 1M (2503024, Gibco), 5 ml Penicillin/Streptomycin (15140122; Gibco), and 1 ml 2-mercaptoethanol (Gibco, 31350-010). In vitro differentiation of the ESCs towards endoderm was carried out in monolayer on 0.1% gelatine coated dishes. The cells were mouse embryo fibroblast feeder cells (MEF) depleted and cultured for few consecutive passages on gelatine and ESC medium. On the day of differentiation, ESCs were seeded (2.8 million cells for 3 days differentiation and 2.1 million cells for 5 days differentiation) on 10 cm gelatine coated dishes directly in endoderm differentiation medium (EDM) consisting of 500 ml Advanced DMEM / F-12 (1×) (Thermo Fisher Scientific; 12634-10), 500 ml Advanced RPMI 1640 (1×) (Thermo Fisher Scientific; 12633-012), 22 ml GlutaMAXTM–I CTSTM (Thermo Fisher Scientific; 12860-01), 200 μl AlbuMAX 100mg/ml (Thermo Fisher Scientific; 11021-029), 22 ml HEPES 1M (Thermo Fisher Scientific; 15630-056), 70 μl Cytidine 150 mg/ml (SIGMA; C4654), 0,9 ml ß-mercaptoethanol 50 mM (Thermo Fisher Scientific; 31350-10), 12 ml Pen/Strep (10 000 U/ml) (Thermo Fisher Scientific; 10378016), 1 ml Insulin-Transferin-Selenium Ethanolamine (Thermo Fisher Scientific; 51500-056), supplemented with 1 ng/ml of murine Wnt3a (1324 WN-CF, R&D systems) and 10 ng/ml of Activin A (338-AC, R&D systems). Freshly prepared EDM supplemented with Wnt3a and Activin A was added every day. Cells were collected on day 3 and day 5 for FACS isolation and routinely tested for mycoplasma contamination.

### Endoderm differentiation of Foxa2^Venus^ ESCs

Prior to endoderm differentiation Wnt3a feeder cells (0.15 × 10^6^/well) (20) were seeded on 0.1% gelatin coated six well plates in endoderm differentiation medium (EDM), consisting of 500 ml advanced DMEM/F-12, 500 ml advanced RPMI, 2.2× GlutaMAX, 20 mg/l Albumax, 22 mM HEPES, 10 μg/ml Cytidine, 0.045 mM ß-mercaptoethanol.

In parallel, the Foxa2-Venus KO ESCs were split on mitomycin-treated MEFs in ESC medium without LIF. The following day, the ESCs (C59 het, C63 het, C9 homo and C17 homo) were pre-plated twice to isolate the ESCs from the mitomycin-treated feeder cells and subsequently 0.6 × 10^6^ ESCs were seeded on the Wnt3a feeders cells in 1:1 Wnt3a conditioned medium and EDM containing Activin A (12 ng/ml). Twenty four hours later, the medium was replaced with EDM containing Activin A (12 ng/ml) and refreshed every day. Cells were collected on day 3 for FACS isolation and routinely tested for mycoplasma contamination.

### RNAseq of DKI cells

Total RNA from two independent biological replicates of day0, day3F+ and day5FS+ was isolated employing the RNA Clean & Concentrator kit (Zymo Research) including digestion of remaining genomic DNA according to producer′s guidelines. The Agilent 2100 Bioanalyzer was used to assess RNA quality and only high quality RNA (RIN > 8) was further processed for removal of ribosomal RNA with the Ribo-Zero Magnetic Gold Kit (Human/Mouse/Rat; Illumina). Ribosomal-depleted RNA was used as input for library preparation with Illumina TruSeq V2 RNA prep kit and processed according to the manufacturer's instruction. Libraries were quality controlled by Qubit and Agilent DNA Bioanalyzer analysis. Deep sequencing was performed on a HiSeq 2500 system according to the standard Illumina protocol for 100 bp paired end reads with v3 sequencing reagents.

### RNAseq of Foxa2^Venus^ and Doxy-Foxa2 cells

Total RNA from FACS-sorted cells was isolated employing RNA Clean & Concentrator kit (Zymo Research) including digestion of remaining genomic DNA according to producer's guidelines. The Agilent 2100 Bioanalyzer was used to assess RNA quality and only high-quality RNA (RIN > 8) was further processed for cDNA synthesis with SMART-Seq v4 Ultra Low Input RNA Kit (Clontech cat. 634888) according to the manufacturer's instruction. cDNA was fragmented to an average size of 200–500 bp in a Covaris S220 device (5 min; 4°C; PP 175; DF 10; CB 200). Fragmented cDNA was used as input for library preparation with MicroPlex Library Preparation Kit v2 (Diagenode, cat. C05010012) and processed according to the manufacturer's instruction. Libraries were quality controlled by Qubit and Agilent DNA Bioanalyzer analysis. Deep sequencing was performed on a HiSeq 1500 system according to the standard Illumina protocol for 50 bp single-end reads with v3 sequencing reagents.

### ChIP-seq of histone modifications

1-2 million FACS-sorted cross-linked cells (1% formaldehyde, 10min RT) were lysed in 100 ul Buffer-B (50 mM Tris–HCl, pH 8.0, 10 mM EDTA, 1%SDS, 1× protease inhibitors -Roche) and sonicated in a microtube (Covaris; 520045) using a Covaris S220 device until most of the DNA fragments were 200–500 base pairs long (settings: temperature 4°C, duty cycle 2%, peak incident power 105 W, cycles per burst 200). After shearing, lysates were centrifuged 10 min, 4°C, 12 000g and supernatant diluted with 900 ul of Buffer-A (10 mM Tris–HCl, pH 7.5, 1 mM EDTA, 0.5 mM EGTA,1% Triton X-100, 0.1% SDS, 0.1% Na-deoxycholate, 140 mM NaCl, 1× protease inhibitors-Roche). 150 ul of sonicated chromatin was then incubated 4 h at 4°C on a rotating wheel with 3 μg of antibody conjugated to 10 μl of Protein G Dynabeads (ThermoFisher). The antibodies used were: anti-H3K4me1 (Diagenode; Pab-037-050), H3K4me3 (Diagenode; Pab-003-050), H3K27ac (Diagenode; Pab-174-050), H3K27me3 (Diagenode; Pab-069-050), H3K9me3 (Diagenode; Pab-056-050), H4K20me3 (Diagenode; Pab-057-050). Beads were washed four times with Buffer-A (10 mM Tris–HCl, pH 7.5, 1 mM EDTA, 0.5 mM EGTA,1% Triton X-100, 0.1% SDS, 0.1% Na-deoxycholate, 140 mM NaCl, 1× protease inhibitors-Roche) and once with Buffer-C (10 mM Tris–HCl, pH 8.0, 10 mM EDTA). Beads were re-suspended in 100 μl elution buffer (50 mM Tris–HCl, pH 8.0, 10 mM EDTA, 1% SDS) and incubated 20 min at 65°C. Supernatant was transferred to a new tube. Crosslink reversal of immunoprecipitated DNA was carried out overnight at 65°C. Then 100 μl TE (10 mM Tris–HCl, pH 8.0, 1 mM EDTA) was added, RNA was degraded by 4 μl RNase A (10 mg/ml) for 1 h at 37°C and proteins were digested with 4 μl Proteinase K (10 mg/ml) at 55°C for 2 h. Finally, DNA was isolated by phenol:chloroform:Isoamyl alcohol purification followed by ethanol precipitation. Purified DNA was used as input for library preparation with MicroPlex Library Preparation Kit v2 (Diagenode, cat. C05010012) and processed according to the manufacturer′s instruction. Libraries were quality controlled by Qubit and Agilent DNA Bioanalyzer analysis. Deep sequencing was performed on HiSeq 1500/2500 systems according to the standard Illumina protocol for 50bp single-end reads using v3 reagents.

### ChIP-seq of transcription factors

1-2 million FACS-sorted cross-linked cells (1% formaldehyde, 10min RT) were lysed in 100 ul Buffer-B-0.3 (50 mM Tris–HCl, pH 8.0, 10 mM EDTA, 0.3% SDS, 1× protease inhibitors -Roche) and sonicated in a microtube (Covaris; 520045) using a Covaris S220 device until most of the DNA fragments were 200–500 base pairs long (settings: temperature 4°C, duty cycle 2%, peak incident power 105 W, cycles per burst 200). After shearing, lysates were centrifuged 10 min, 4°C, 12 000g and supernatant diluted with 1 volume of Dilution Buffer (1 mM EGTA 300 mM NaCl, 2% Triton X-100, 0.2% sodium deoxycholate, 1× protease inhibitors-Roche). Sonicated chromatin was then incubated 4 h at 4°C on a rotating wheel with 6 ug of antibody conjugated to 20 μl of Protein G Dynabeads (ThermoFisher). The antibodies used were anti-Foxa2 (SantaCruz; sc6554x), anti-Gata4 (R&D Systems; AF2606), anti-Nanog (Bethyl lab; A300-397-A). Beads were washed four times with Buffer-A (10 mM Tris–HCl, pH 7.5, 1 mM EDTA, 0.5 mM EGTA,1% Triton X-100, 0.1% SDS, 0.1% Na-deoxycholate, 140 mM NaCl, 1× protease inhibitors) and once with Buffer-C (10 mM Tris–HCl, pH 8.0, 10 mM EDTA). Beads were then incubated with 70 μl elution buffer (0.5% SDS, 300 mM NaCl, 5 mM EDTA, 10 mM Tris–HCl pH 8.0) containing 2 μl of Proteinase K (20 mg/ml) for 1 h at 55°C and 8 h at 65°C to revert formaldehyde crosslinking, and supernatant was transferred to a new tube. Another 30 μl of elution buffer was added to the beads for 1 min and eluates were combined and incubated with another 1 μl of Proteinase K for 1 h at 55°C. Finally, DNA was purified with SPRI AMPure XP beads (Beckman Coulter) (sample-to-beads ratio 1:2). Purified DNA was used as input for library preparation with MicroPlex Library Preparation Kit v2 (Diagenode, cat. C05010012) and processed according to the manufacturer′s instruction. Libraries were quality controlled by Qubit and Agilent DNA Bioanalyzer analysis. Deep sequencing was performed on HiSeq 1500/2500 systems according to the standard Illumina protocol for 50 bp single-end reads using v3 reagents.

### meDIP-seq and Hydroxy-meDIP-seq

The procedure was adapted from (21,22). Genomic DNA from FACS-sorted cells was randomly sheared to 100–500 bp in a microtube (Covaris; 520045) using a Covaris S220 device (400 s; 4°C; PP 140; DF 10; CB 200). Sonicated DNA was end-repaired, A-tailed and ligated to Illumina multiplex adaptors according to NEBNext DNA library prep kit (NEB E6040S). Ligated DNA was purified using Agencourt AMPure XP beads (Beckman Coulter). 1 μg of adaptor-ligated DNA was used for each immunoprecipitation and heat-denatured at 95°C for 10 min, rapidly cooled on ice and immunoprecipitated overnight at 4°C with rocking agitation in 500 ml immunoprecipitation buffer (10mM sodium phosphate buffer, pH 7.0, 140mM NaCl, 0.05% Triton X-100) using 1 μl of mouse monoclonal anti-5-methylcytosine antibody (Eurogentec BI-MECY-0100) or 0,5 μl of rabbit 5-Hydroxymethylcytosine antibody (Active Motif 39769). To recover the antibody-bound DNA fragments, 50 μl Protein G Dynabeads (ThermoFisher) and, only to anti-5-methylcytosine IPs, 5 μl of rabbit anti-mouse IgG secondary antibody (Active Motif 53017) were added and incubated for an additional 2 h at 4 °C with agitation. After immunoprecipitation a total of 7–10 immunoprecipitation washes were performed with ice-cold immunoprecipitation buffer. Washed beads were resuspended in TE buffer with 0.25% SDS and 0.25 mg/ml proteinase K for 2 h at 55 °C with vigorous shaking (900 rpm). DNA was purified with the PCR clean-up MinElute kit (Qiagen) and eluted in 30 ul. Samples were then amplified by PCR with Illumina primers (NEBNext Multiplex Oligos for Illumina cat. E7335) in a 50 μl reaction with 2× PCR master mix (NEB cat. M0541). PCR cycled as: (i) 98°C, 30 s; (ii) 98°C, 10 s; (iii) 60°C, 30 s; (iv) 72°C, 30 s; (v) repeat steps (ii)–(iv) for 4–10 cycles; (vi) 72°C, 5 min. Amplified libraries were purified using Agencourt AMPure XP beads (Beckman Coulter). Quality control was carried out with a Qubit fluorometer and a Bioanalyzer (Agilent). 50 bp single-end sequencing was performed with a a HiSeq 1500 sequencer with v3 reagents (Illumina).

### ATAC-seq

ATAC-seq was done as previously described (23). Briefly, 50 000 FACS sorted cells were washed in 1× PBS, re-suspended in 50 ul of lysis buffer (10 mM Tris pH 7.4, 10 mM NaCl, 3 mM MgCl_2_, 0.1% NP40,) and spun at 500 g for 10 min at 4°C to collect nuclei. Nuclei were subsequently re-suspended in 50 μl Transposase reaction containing 25 μl 2× tagmentation buffer, 22.5 μl water, 2.5 μl Tn5 Transposase (Illumina Nextera DNA Library Preparation Kit, cat. FC-121-1030). Reactions were incubated for 30 min at 37°C in a thermomixer shaking at 300 rpm and DNA purified using PCR clean-up MinElute kit (Qiagen). The transposed DNA was subsequently amplified in 50 μl reactions with custom primers as described (23). After 4 cycles libraries were then monitored with qPCR: 5 μl PCR sample in a 15 μl reaction with the same primers. qPCR output was monitored for the ΔRN; 0.25 ΔRN cycle number was used to estimate the number of additional cycles of the PCR reaction needed for the remaining PCR samples. Amplified libraries were purified with the PCR clean-up MinElute kit (Qiagen) and size selected for fragments <600 bp using the Agencourt AMPure XP beads (Beckman Coulter). Libraries were quality controlled by Qubit and Agilent DNA Bioanalyzer analysis. Deep sequencing was performed on a HiSeq 1500 system according to the standard Illumina protocol for 50 bp single-end or paired-end reads.

### Fluorescence activated cell sorting

For RNAseq, ATACseq and meDIP-seq, following trypsin treatment, cells were resuspended in PBS with 10% FCS before FACS collection. For ChIP-seq cells, cells were fixed for 10 min with 1% formaldehyde and quenched with 0.125 M final concentration glycine before FACS collection. FACS was performed with a FACSAria instrument (BD Biosciences). Data were analyzed with FlowJo software.

## RESULTS

### Isolation of Foxa2 expressing mesendoderm and endoderm cells

**Figure 1.**
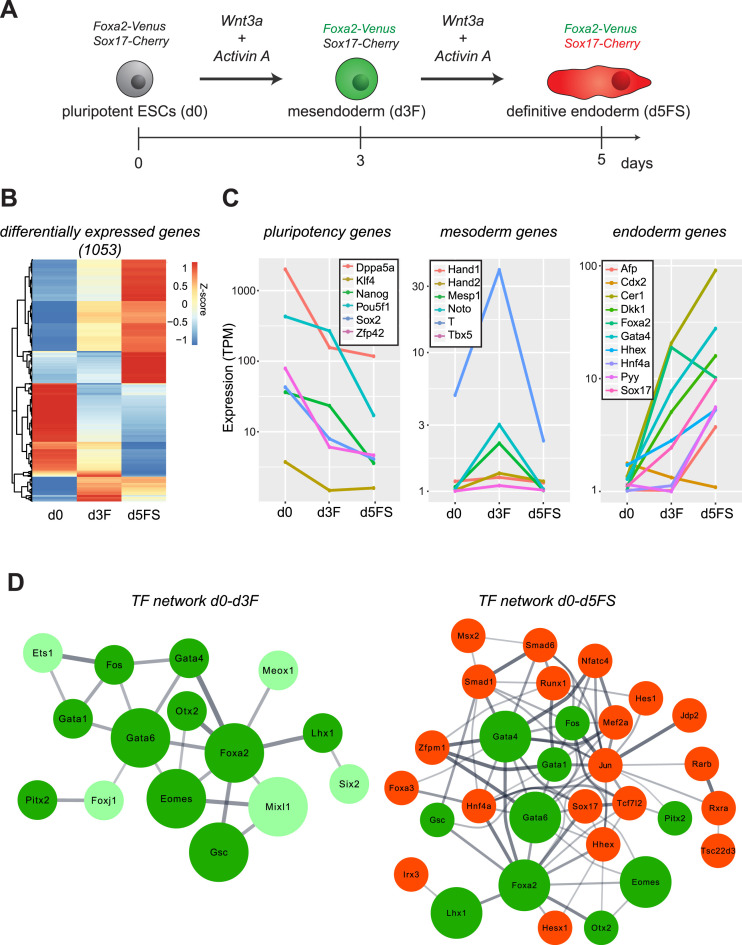
An in vitro differentiation system modelling early endoderm differentiation. (**A**) Endoderm differentiation of ESCs is triggered by Wnt3A/Activin A treatment. Mesendoderm (Foxa2-Venus^pos^; Sox17-Cherry^neg^) and endoderm (Foxa2-Venus^pos^; Sox17-Cherry^pos^) cells can be isolated by FACS. (**B**) Heat map showing z-scores of the expression levels of the 1053 differentially expressed genes between pluripotent ESCs (d0), mesendoderm (d3F) and endoderm (d5FS) cells (*P*_adj_ < 0.05, fold change > 2; *n* = 2 for each condition). (**C**) Average expression levels of selected marker genes for pluripotent, mesoderm and endoderm cells in the *in vitro* differentiated ESCs (TPM – Transcripts Per Kilobase Million; *n* = 2 for each condition). (**D**) Network of most influential transcription factors, driving transition from pluripotent ESCs (d0) through a mesendoderm stage (d3F) to definitive endoderm (d5FS) cells. Bigger nodes correspond to the top 5 transcription factors. Width of edges corresponds to String database (StringDB) scores. Only connected nodes are plotted. Color code: light green factors are specific to the d0–d3F network, green factors are present in both networks, red factors emerge in the d0–d5FS network.

To study Foxa2 binding site selection and Foxa2-dependent chromatin changes we decided to investigate the transition from pluripotent ESCs via mesendoderm (MESEND) progenitors to definitive endoderm (DE) cells using an *in vitro* differentiation system (Figure 1A). For isolating cells from specific stages of endoderm commitment in high purity we made use of a double knock-in (DKI) mouse ESC line carrying Foxa2-Venus and Sox17-Cherry fusion reporter genes (18,19). The resulting TF fluorescent fusion proteins are expected to be functional, as homozygous *Foxa2^FVF/FVF^; Sox17^SCF/SCF^* mice are fully viable without any obvious phenotypes. By fluorescence-activated cell sorting (FACS) we isolated pure populations of Foxa2-Venus^neg^/Sox17-Cherry^neg^ pluripotent ESCs, Foxa2-Venus^pos^/Sox17-Cherry^neg^ mesendoderm cells after three days and Foxa2-Venus^pos^/Sox17-Cherry^pos^ cells after five days of differentiation (Figure 1A, Supplementary Figure S1A). We refer to these cell populations as d0 (pluripotent ESCs), d3F (Foxa2^pos^ mesendoderm cells) and d5FS (Foxa2^pos^/Sox17^pos^ definitive endoderm cells). Transcriptome analyses of these populations revealed stage-specific expression signatures with 1053 differentially expressed genes (fold change > 2; *P*_adj_ < 0.05; Figure 1B, Supplementary Figure S1B, C, Supplementary Table S1). Consistent with the progressive differentiation to endoderm we observed downregulation of pluripotency genes, a transient expression of mesoderm genes in d3F cells and progressive induction of endoderm genes in d3F and d5FS cells (Figure 1C). Expression of anterior endoderm markers (Cer1, Dkk1) and absence of posterior definitive endoderm markers (Cdx2) suggests that the *in vitro* differentiation favors the generation of cells resembling anterior definitive endoderm. To highlight the transcription factor network responsible for endoderm differentiation we identified the most influential transcription factors by their expression change and connectivity (Figure 1D). This analysis suggests that transcription factors such as Foxa2, Gata4 and Eomes appear as most important to initiate endoderm differentiation (d0–d3F network), while the importance of additional TFs emerges in later stages of endoderm differentiation (d0–d5FS network).

Thus, by combining *in vitro* endoderm differentiation with FACS sorting we could isolate two consecutive stages of endoderm differentiation resembling features of mesendoderm and anterior definitive endoderm cells.

### Foxa motifs are over-represented in regions of increased chromatin accessibility upon endoderm differentiation

**Figure 2.**
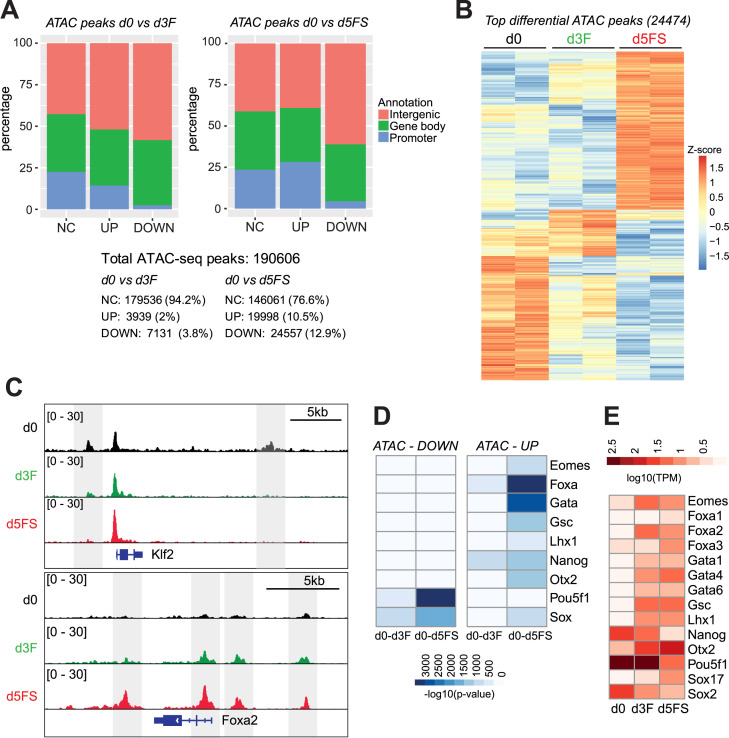
Foxa motifs are over-represented in regions of increased chromatin accessibility upon endoderm differentiation. (**A**) Numbers and percentages of not changed (NC) and dynamic (UP/DOWN) ATAC-seq peaks associated with different genomic features in d0 versus d3F (left panel) or in d0 versus d5FS (right panel) comparison. Peaks are considered dynamic with an ATAC-seq coverage fold change >2. (**B**) Heat map showing relative chromatin accessibility (*z*-scores of normalized ATAC-seq signals) of the top dynamic ATAC-seq peaks (24 474) in pluripotent ESCs (d0), mesendoderm (d3F) and endoderm (d5FS) cells (*P*_adj_ < 0.05, fold change > 4; *n* = 2 for each condition). (**C**) Representative genome browser view of ATAC-seq signals in d0, d3F and d5FS cells. (**D**) Heat map showing the *P*-values of transcription factor motif enrichments in dynamic ATAC-seq peaks. The Homer tool was used to scan for known motifs of expressed transcription factors (TPM > 1). Only the top scoring motifs with –log10 (*P*-value) > 500 are shown. Since members of Foxa, Gata and Sox families bind very similar motifs only the family names are given. Columns represent analyses for differential ATAC peaks between d0–d3F and d0–d5FS. (**E**) Expression heatmap of transcription factors shown in (D) in d0, d3F and d5FS cells. Relevant Foxa, Gata and Sox family members are shown.

To get additional insight into the gene regulatory network governing the transition from mouse pluripotent ESCs via MESEND progenitors to the DE stage we used the assay of transposase-accessible chromatin using sequencing (ATAC-seq) (23) to determine the genome-wide chromatin accessibility landscape in d0, d3F and d5FS cells. Overall, we identified 190606 accessible regions, located primarily at non-promoter regions representing putative enhancers (Supplementary Figure S2A). Consistent with previous reports (24,25), the PCA analysis of all ATAC peaks, promoter peaks or non-promoter peaks demonstrated that in particular non-promoter ATAC peaks are a strongly distinguishing feature of the three cell populations (Supplementary Figure S2C). We then assessed differential accessibility between the differentiation stages (fold change > 2, P_adj_ < 0.05) and found that 5.8% ATAC peaks change in the transition d0–d3F and 23.4% in the transition d0–d5FS (Figure 2A–C). The differentially accessible regions (DARs) are located primarily at non-promoter regions (Figure 2A). A heatmap of the top regulated ATAC peaks during endoderm differentiation (Figure 2B) recapitulates the pattern of transcriptional changes observed by RNA-seq (Figure 1B). Thus, DARs show a good correlation with regulated genes (Supplementary Figure S2B), although the analysis is limited by connecting individual ATAC peaks with specific genes only by proximity to the TSS. To enhance the biological insights obtained from DARs we analysed the annotations of the nearby genes with the GREAT tool (26). In particular, peaks that change in the d0–d3F transition are associated with gene ontology annotations connected to loss of stem cell properties and the emergence of differentiated cells (Supplementary Figure S2D-F, Supplementary Table S2).

Next, to identify transcription factors responsible for establishing DARs during endoderm differentiation we determined DNA binding motifs within differential ATAC peaks (Supplementary Table S3). We found that DARs with reduced accessibility (DOWN) are mostly enriched for motifs of pluripotency related TFs, such as Oct4 (Pou5f1) and Sox family factors (Figure 2D, E), consistent with downregulation of the pluripotency network. In contrast, DARs with increased accessibility (UP) are mostly enriched with motifs of mesendoderm- and endoderm-related TFs, such as Foxa family, Gata family, Eomes, Lhx1, Otx2 and Gsc (Figure 2D, E). Notably, upregulated DARs most prominently feature Foxa motifs. Based on the expression of the Foxa family members (Figure 2E) and the fact that Foxa2 is expressed first among the Foxa family (27) we hypothesize that the PTF Foxa2 is a key factor to induce chromatin accessibility in the context of endoderm differentiation.

### Loss of Foxa2 impairs endoderm differentiation

Foxa2 knock-out mice show early embryonic lethality (E9–E10) and absence of anterior definitive endoderm and axial mesoderm (28,29), indicating its functional importance for mesendoderm and endoderm development. To investigate if Foxa2 is also critical for *in vitro* endoderm differentiation, we generated a Foxa2 knock-in/knock-out allele (Foxa2^Venus^) by replacing the coding region of Foxa2 with an H2B-Venus expression cassette in mouse ESCs (Supplementary Figure S3A–C). As H2B-Venus is under control of the Foxa2 promoter we detected nuclear H2B-Venus protein only upon mesendoderm differentiation in both control (Foxa2^Venus/+^) and Foxa2 ko (Foxa2^Venus/Venus^) ESCs, suggesting that initiation of Foxa2 expression is independent of Foxa2 protein (Supplementary Figure S3D).

**Figure 3.**
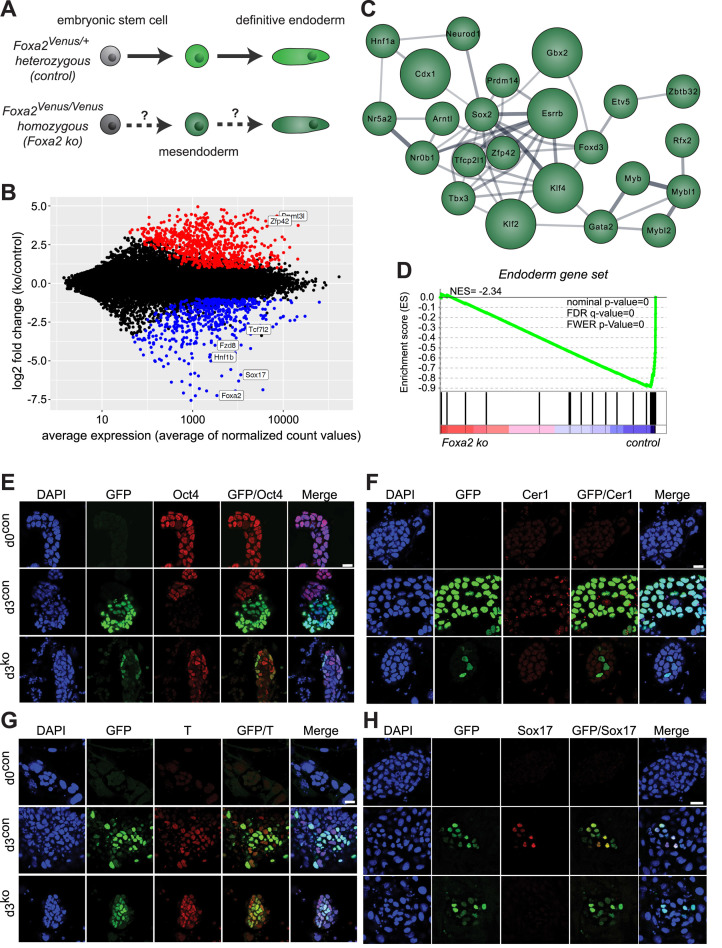
Loss of FOXA2 impairs endoderm differentiation. (**A**) Differentiation and FACS sorting strategy of control versus Foxa2 ko ESC into endoderm. (**B**) Dotplot showing average expression versus log_2_-fold change of coding genes in endoderm differentiating Foxa2^Venus/+^ control (d3^con^) versus Foxa2^Venus/Venus^ ko (d3^ko^) cells. Coloured dots indicate genes with significantly changed expression (P_adj_ < 0.05, fold change > 2; *n* = 3 for each condition). Positions of relevant genes are indicated. (**C**) Network of most influential transcription factors in endoderm differentiating Foxa2^Venus/Venus^ ko (d3^ko^) cells. Bigger nodes correspond to the top 5 transcription factors. Width of edges corresponds to String database (StringDB) scores. The network has no overlap with the one shown in Figure 1D. (**D**) Gene set enrichment analysis (GSEA) of an endoderm gene set between control (d3^con^) and Foxa2 ko cells (d3^ko^). The Foxa2 ko cells show strong underrepresentation of these endoderm signature genes. NES: normalized enrichment score. (**E**) Confocal sections showing undifferentiated Foxa2^Venus/+^ (d0^con^), endoderm differentiating Foxa2^Venus/+^ (d3^con^) and Foxa2^Venus/Venus^ homozygous (d3^ko^) cells stained with antibodies to Venus/GFP (green), Oct4 (red) and DAPI (blue). Scale bar: 20 μm. (**F**) Confocal sections showing undifferentiated Foxa2^Venus/+^ (d0^con^), endoderm differentiating Foxa2^Venus/+^ (d3^con^) and Foxa2^Venus/Venus^ (d3^ko^) cells stained with antibodies to Venus/GFP (green), Cer1 (red) and DAPI (blue). Scale bar: 20 μm. (**G**) Confocal sections showing undifferentiated Foxa2^Venus/+^ (d0^con^), endoderm differentiating Foxa2^Venus/+^ (d3^con^) and Foxa2^Venus/Venus^ (d3^ko^) cells stained with antibodies to Venus/GFP (green), Brachyury/T (red) and DAPI (blue). Scale bar: 20μm. (**H**) Confocal sections showing undifferentiated Foxa2^Venus/+^ (d0^con^), endoderm differentiating Foxa2^Venus/+^ (d3^con^) and Foxa2^Venus/Venus^ (d3^ko^) cells stained with antibodies to Venus/GFP (green), Sox17 (red) and DAPI (blue). Scale bar: 20μm.

Next, we investigated Foxa2-dependent transcriptional changes. We induced endoderm differentiation in both control and Foxa2 ko ESCs and FACS-isolated Venus-positive cells at day 3 of differentiation (Figure 3A, Supplementary Figure S3E). We then performed RNA-seq based transcriptome analysis of undifferentiated (control = d0^con^; ko = d0^ko^) and differentiating (control = d3^con^, ko = d3^ko^) cells. In agreement with the fact that Foxa2 is not expressed in ESCs we did not observe transcriptional differences between d0^con^ and d0^ko^ cells, however, differentiating d3^con^ and d3^ko^ cells were clearly distinct (Supplementary Figure S3F). We identified 1268 differentially expressed genes (fold change > 2, *P*_adj_ < 0.01) between d3^con^ and d3^ko^ cells (Figure 3B, Supplementary Table S4). Gene ontology analysis of these genes showed a dominant enrichment for terms associated with embryonic development (Supplementary Figure S3G). We found that in d3^ko^ cells pluripotency genes were not properly downregulated and endoderm genes were not fully activated, while mesoderm genes did not show obvious changes (Supplementary Figure S3H–J). These findings reflect downregulation of an endoderm signature gene set (30) in d3^ko^ cells (Figure 3D). The failure to differentiate to endoderm is likely to be linked with the aberrant transcription factor network of d3^ko^ cells (Figure 3C), which has no overlap with the endoderm differentiation networks observed in d3F and d5FS cells (Figure 1D).

Consistent with the transcriptional changes we detected sustained levels of Oct4 protein d3^ko^ cells (Figure 3E). Mesendoderm marker gene Brachyury (T) was comparable between d3^con^ and d3^ko^ cells (Figure 3G), whereas key endoderm TFs and signaling factors, such as Cer1 and Sox17, were not induced in d3^ko^ cells (Figure 3F, H). In summary, our expression analyses show that Foxa2 ko cells are not able to fully activate the endoderm program which likely results in appropriate downregulation of important pluripotency genes. Taken together, these data demonstrate that Foxa2 is a master regulator for endoderm differentiation in ESC *in vitro* differentiation comparable to its *in vivo* function (31).

### Foxa2 binding sites are dynamic during endoderm differentiation.

**Figure 4.**
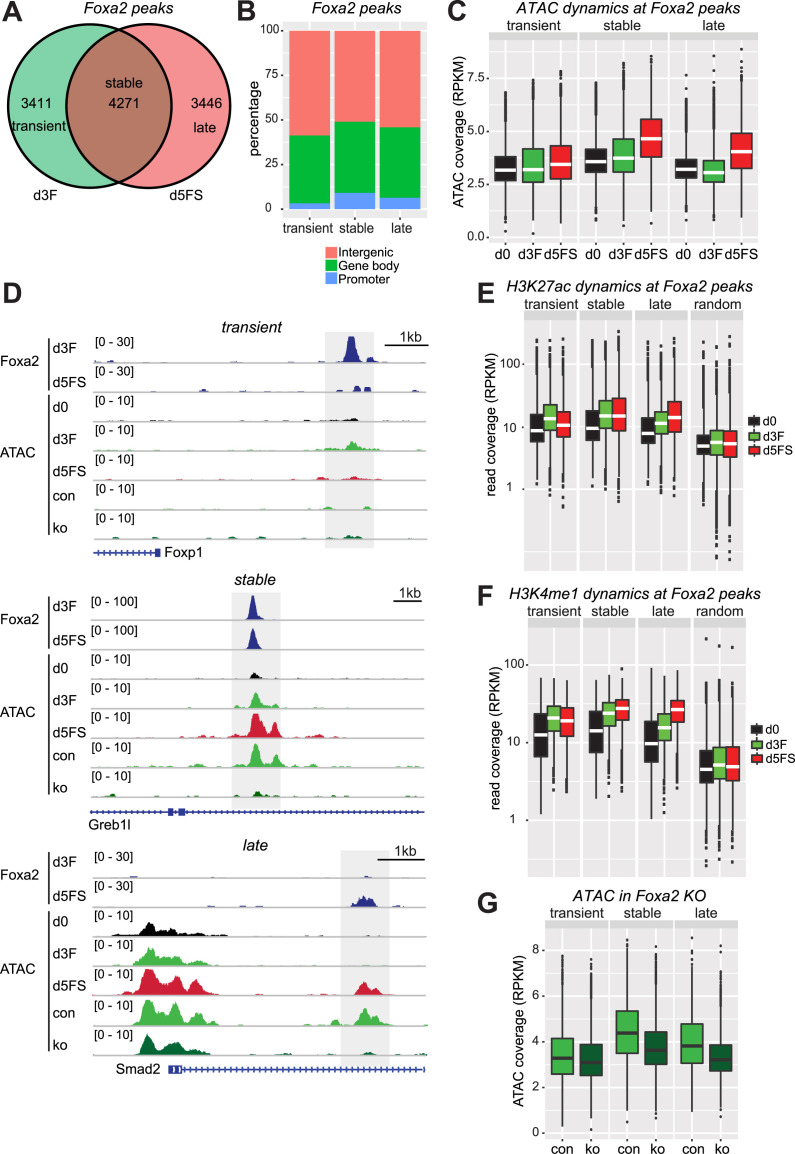
Foxa2 is required for chromatin opening. (**A**) Venn diagram of Foxa2 binding sites in d3F (green) and d5FS (red) cells. d3F- and d5FS-specific binding sites were assigned ‘transient’ and ‘late’, respectively; overlapping binding sites were assigned ‘stable’. *n* = 2 for each condition. (**B**) Percentages of transient, stable and late Foxa2 binding site associated with different genomic features. (**C**) Box plot of normalized ATAC-seq coverage on transient, stable and late Foxa2 binding sites in d0, d3F and d5FS cells. *n* = 2 for each condition. (**D**) Genome browser view of examples for transient, stable and late Foxa2 binding sites. The following tracks are displayed: Foxa2 ChIP-seq in d3F and d5FS cells; ATAC-seq in d0, d3F, d5FS, Foxa2^Venus/+^ (d3^con^) and Foxa2^Venus/Venus^ (d3^ko^) endoderm differentiating cells. Dashed regions indicate Foxa2 binding sites. (**E**) Box plot of normalized H3K27ac ChIP-seq coverage on Foxa2 binding sites and random genomic regions in d0, d3F and d5FS cells. *n* = 2 for each condition.(**F**) Box plot of normalized H3K4me1 ChIP-seq coverage on Foxa2 binding sites and random regions in d0, d3F and d5FS cells. *n* = 2 for each condition. (**G**) Box plots of normalized ATAC-seq coverage on transient, stable and late Foxa2 binding sites in endoderm differentiating Foxa2^Venus/+^ (d3^con^) and Foxa2^Venus/Venus^ (d3^ko^) cells isolated at day 3 of differentiation. *n* = 3 for each condition. Wilcoxon ranks-sum test statistics for all the box plots is shown in Supplementary Table S7.

To gain better insight into the fundamental roles of Foxa2 for endoderm differentiation we mapped Foxa2 binding sites by chromatin immunoprecipitation followed by next generation sequencing (ChIP-seq) in d3F and d5FS cells. We made use of a specific Foxa2 antibody (32) and considered only those sites common to two replicates as high confidence binding sites. Interestingly, Foxa2 binding is highly dynamic between the differentiation states. We identified 3411 binding sites specific for d3F cells, 4271 binding sites which are shared between d3F and d5FS and 3446 binding sites specific to d5FS cells (Figure 4A, D, Supplementary Table S5). We named these categories of binding sites ‘transient’, ‘stable’ and ‘late’, respectively. All binding categories display prominent presence of the Foxa DNA motif (Supplementary Figure S4A, Supplementary Table S6), demonstrating specificity of the ChIP experiment. Consistent with previous reports (33,34) we observed the majority of Foxa2 binding sites located at non-promoter regions (Figure 4B), suggesting that Foxa2 is primarily involved in gene regulation through distal cis-regulatory regions. Furthermore, to gain insights into the biological function of the Foxa2 binding sites, we analysed the annotations of the nearby genes with the GREAT tool (26). All the three categories of Foxa2 binding sites are enriched for gene ontology annotations associated to differentiation and development (Supplementary Figure S4B). Stable and late Foxa2 binding sites are also flanked by genes of the Foxa network, but only stable binding sites are enriched for genes of the Wnt pathway (Supplementary Figure S4C), suggesting a diversified biological function for the different Foxa2 binding sites. Next we aimed for correlating Foxa2 binding with gene expression changes. Due to the large number of Foxa2 binding sites not all these binding events are likely to cause changes in gene expression, as also observed for other transcription factors (35). However, we found that a large percentage (∼30%) of genes that change expression over the time course or in Foxa2 ko cells are bound by Foxa2 (Supplementary Figure S4D, E), suggesting that Foxa2 is important for regulating their expression and consistent with the important role of Foxa2 for endoderm development.

Taken together, our data show that Foxa2 binding is highly dynamic during endoderm differentiation, with transient, stable and late binding sites in the vicinity of key developmental genes.

### Foxa2 is required for chromatin opening and recruitment of active histone modifications

Being a pioneer factor, Foxa2 is expected to mediate chromatin opening (17) or nucleosome depletion (6,16) on its binding sites. We wondered if the different categories of Foxa2 binding sites show differences in these respects and functions. Thus, we analysed ATAC-seq coverage on transient, stable and late Foxa2 binding sites as a proxy for chromatin accessibility. Remarkably, we detected major differences in chromatin accessibility (Figure 4C, D, Supplementary Table S7). In stable and late Foxa2 binding sites we could observe increased chromatin accessibility mainly in d5FS cells (Figure 4C, D, Supplementary Figure S4G–I). In contrast, transient Foxa2 binding sites showed almost no change in chromatin accessibility (Figure 4C, D, Supplementary Figure S4F, I). These data demonstrate that binding of Foxa2 in d3F cells does not lead to increased chromatin accessibility at most of its binding sites.

Since Foxa2 binding did not fully correlate with increased chromatin accessibility, we asked if Foxa2 recruitment would better correlate with changes in enhancer chromatin modifications (36). For both, H3K4me1 and H3K27ac, we observed increased levels on stable and late binding sites during endoderm differentiation (Figure 4E, F, Supplementary Table S7). Enhancer modifications were also increased in d3F cells on transient binding sites, however, loss of Foxa2 from these binding sites in d5FS cells correlated with reduced levels of these modifications (Figure 4E, F, Supplementary Figure S4J). These data demonstrate that Foxa2 binding strictly correlates with establishment of enhancer chromatin modifications, but only on a subset of binding sites, increased chromatin accessibility can be induced.

To understand if increased chromatin accessibility depends on Foxa2 binding we performed ATAC-seq in Foxa2 deficient cells. As Foxa2^Venus^ ko cells do not carry the *Sox17^SCF^* allele we were not able to isolate specific populations corresponding to d3F and d5FS, but rather FACS-isolated endoderm differentiating cells based on Foxa2 expression. Comparing control (Foxa2^Venus/+^) with Foxa2 ko (Foxa2^Venus/Venus^) cells, we detected reduced chromatin accessibility on stable and late Foxa2 binding sites, whereas no substantial differences could be observed on transient binding sites (Figure 4D, G, Supplementary Table S7, Supplementary Figure S4F–I). In summary our data indicate that Foxa2 is required but not sufficient for chromatin opening at its binding sites.

### Co-binding of Foxa2 with other TFs correlates with chromatin opening

**Figure 5.**
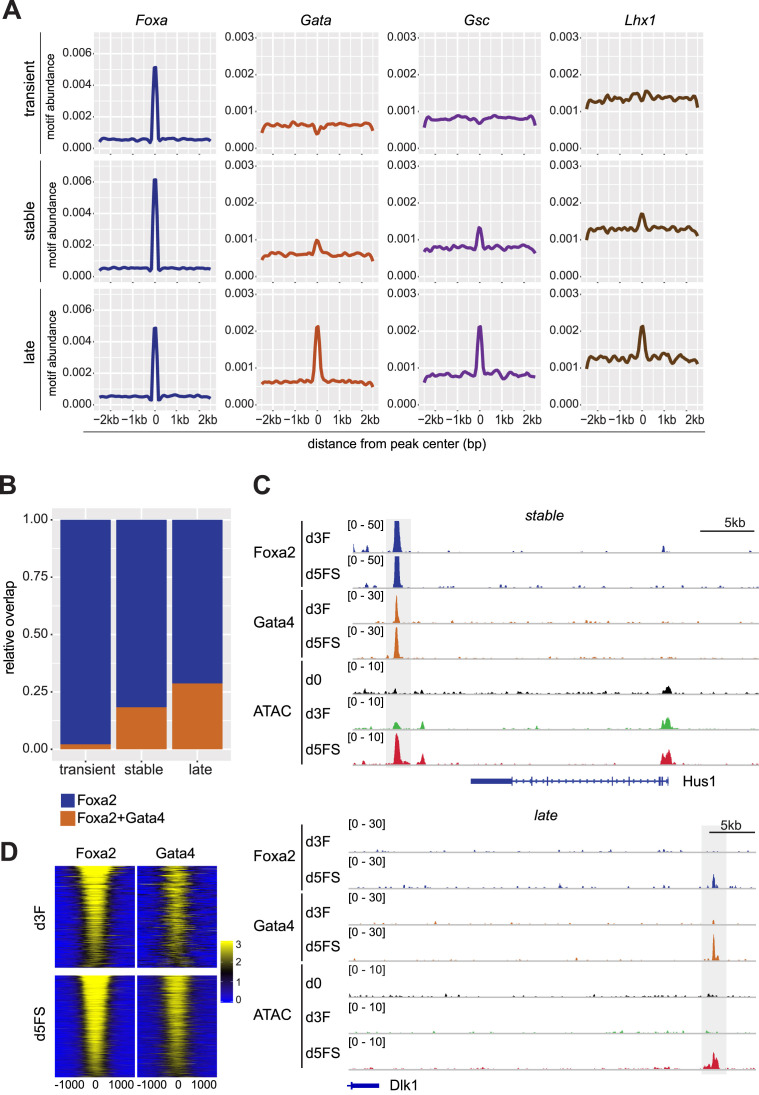
Co-binding of Foxa2 with other TFs correlates with chromatin opening (**A**) Density plots for motif abundances of Foxa, Gata, Gsc and Lhx1 motifs in transient, stable and late Foxa2 binding sites. (**B**) Fraction of transient, stable and late Foxa2 binding sites bound by Foxa2 (blue) or co-bound by Foxa2 and Gata4 (brown). (**C**) Genome browser view of example stable and late Foxa2 binding sites. The following tracks are displayed: Foxa2 and Gata4 ChIP-seq in d3F and d5FS cells, ATAC-seq in d0, d3F and d5FS cells. Dashed regions indicate stable (upper panel) and late (lower panel) Foxa2 binding sites. (**D**) Read-density heat map showing the normalized coverage of Foxa2 and Gata4 on Foxa2 binding sites. Top panel: Foxa2 and Gata4 ChIP-seq in d3F cells. Bottom panel: Foxa2 and Gata4 ChIP-seq in d5FS cells. Distance from the peak centre is given in bp.

We found that binding of Foxa2 to a target locus is not sufficient to induce chromatin accessibility. Therefore, we hypothesized that co-binding of additional proteins, probably other transcription factors, may favour chromatin accessibility. To assess this hypothesis, we analysed the presence of endoderm TF binding motifs at Foxa2 binding sites. As expected, the Foxa motif is strongly enriched in transient, stable and late binding sites (Figure 5A, Supplementary Table S6). Motifs of other mesendoderm- and endoderm-related TFs, such as Gata family, Lhx1 and Gsc, tend to be enriched on stable and late Foxa2 binding sites which display increased chromatin accessibility (Figure 5A). Transient binding sites at which Foxa2 fails to induce chromatin accessibility showed no enrichment for these additional TF binding sites. The presence of a binding motif is not necessarily predictive of actual binding. Thus, we generated ChIP-seq profiles for Gata4, a PTF (7,37) and a prominently expressed member of the Gata family, in d3F and d5FS cell populations. Consistent with the motif predictions our analysis revealed that Gata4 peaks coincide with Foxa2 peaks preferentially at stable and late Foxa2 binding sites (Figure 5B–D, Supplementary Figure S5A). Most of these binding sites display strongly increased chromatin accessibility during endoderm differentiation (Supplementary Figure S5B). Collectively, these data support a model wherein cooperative TF binding and activity is necessary to induce chromatin opening.

### Endoderm-specific Foxa2 binding sites feature active chromatin modifications in ESCs

**Figure 6.**
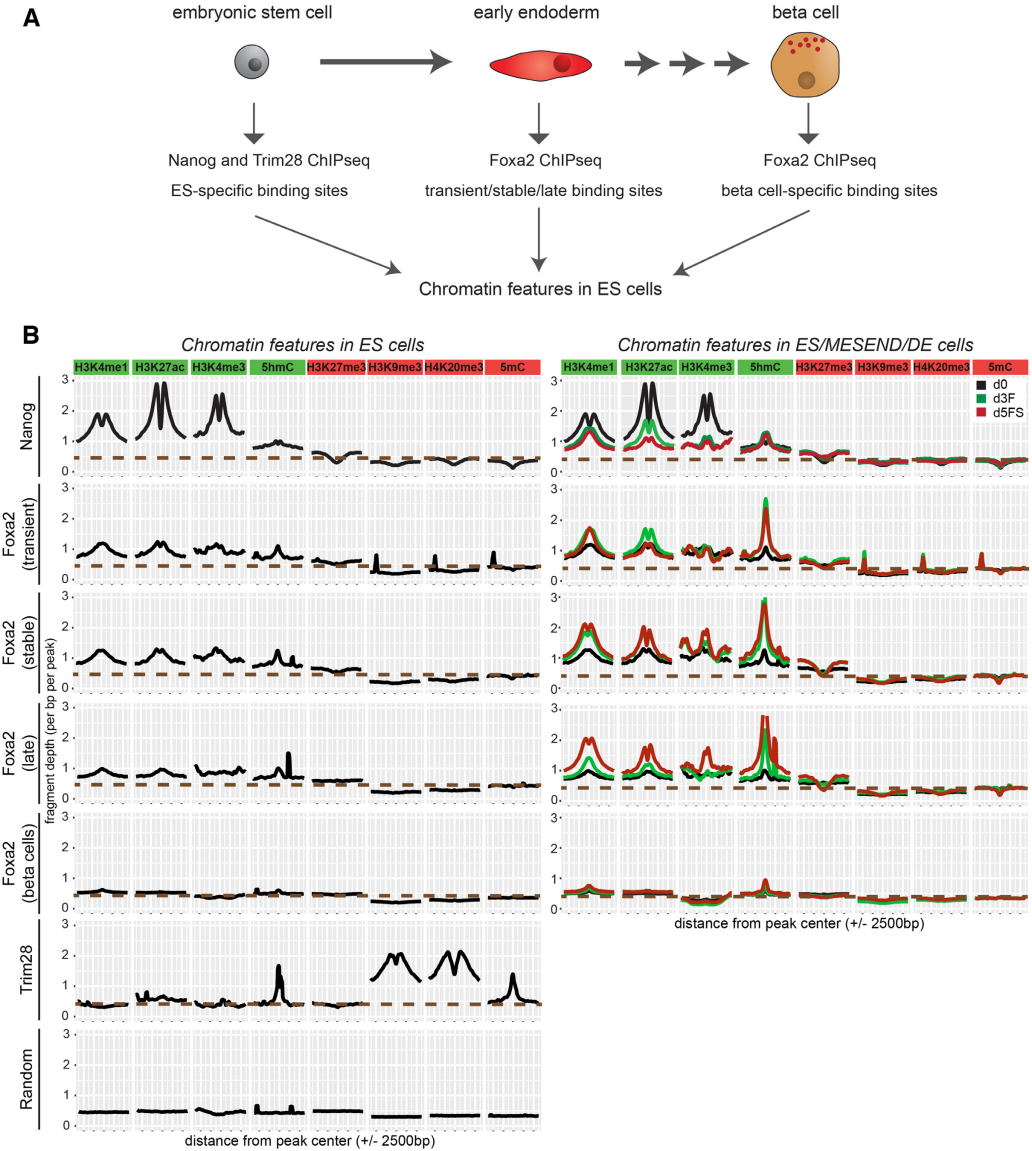
Endoderm-specific Foxa2 binding sites feature active chromatin modifications in ES cells. (**A**) Embryonic stem cells can differentiate in definitive endoderm cells which represent an early stage of endoderm development. Later in development, the pancreas is formed as an endoderm-derived organ that contains insulin-secreting beta cells. We analysed whether Foxa2 binding sites in transient/stable/late or pancreatic beta cells show a different chromatin profile already before Foxa2 expression, in ESCs. As controls for active and repressed region-associated factors, we analysed binding sites of Nanog (active TF) and Trim28 (repressor). (**B**) Left panel: Density plots showing average levels of active (H3K4me1, H3K4me3, H3K27ac, 5hmC) and repressive (H3K27me3, H3K9me3, H4K20me3, 5mC) chromatin modifications in pluripotent ES cells at specific peak sets: Nanog binding sites in ES cells, Foxa2 transient, stable, late binding sites, Foxa2 binding sites in pancreas (beta cells), Trim28 binding sites in ES cells and random genomic regions. Right panel: density plots for active and repressive chromatin modifications in d0 (black), d3F (green) and d5FS (red) cells at Nanog and Foxa2 binding sites.

Foxa2 is continuously expressed during endoderm differentiation in endoderm, pancreatic and liver progenitors as well as in differentiated insulin-producing beta cells (27). However, Foxa2 only binds a subset of its potential binding sites and clear binding differences exist between cell types (8). As chromatin environment could influence transcription factor binding (12–14), we thought to determine chromatin modifications in ESCs which might distinguish endodermal from other Foxa2 binding sites bound at later stages during differentiation. For this analysis we investigated endodermal Foxa2 binding sites (transient, stable and late) compared with pancreatic beta cell-specific binding sites from a published dataset (38). For comparison we defined Nanog binding sites representative of active regulatory regions, Trim28 binding sites (39) corresponding to repressed chromatin, and random genomic regions (Figure 6A).

We investigated by ChIP-seq active (H3K4me1, H3K4me3, H3K27ac) and repressive (H3K9me3, H3K27me3, H4K20me3) histone marks. Further, we analyzed DNA methylation (5mC) and hydroxymethylation (5hmC) by meDIP-seq. Remarkably, endoderm and beta cell-specific binding sites show a distinct chromatin signature in ESCs (Figure 6B, Supplementary Figure S6A–H). Active modifications are selectively present on endoderm-specific binding sites, although at much lower levels as compared to Nanog binding sites. Beta cell-specific binding sites lack these active modifications, but also do not show prominent enrichment of repressive marks (Supplementary Figure S6H, Supplementary Table S7). Heatmap representations of our data demonstrate that active chromatin modifications are detectable on the majority of transient and stable Foxa2 binding sites and at a somewhat lower level on late binding sites (Supplementary Figure S6D–G). During endoderm differentiation, active histone modifications on endodermal Foxa2 binding sites were further elevated, whereas no change was observed on beta cell binding sites (Figure 6B).

In summary, these data suggest that Foxa2 preferentially binds to regions of slightly active chromatin. Repressive modifications, in contrast, were largely absent in all Foxa2 binding sites (Figure 6B, Supplementary Figure S6H). Notably, while re-analysing published datasets on endoderm differentiation of human ES cells (8,40–41), we also observed higher levels of active chromatin marks on endoderm-specific versus liver-specific FOXA2 binding sites (Supplementary Figure S6I, Supplementary Table S7), suggesting that binding preferences are evolutionarily conserved.

### Transcriptional and epigenetic effects of Foxa2 and Gata4 binding in ESCs

Our data suggest that chromatin in ESCs is prepared to favour Foxa2 binding to endoderm-specific binding sites. However, Foxa2 recruitment could be modulated by collaborating endoderm transcription factors (8). Thus, we wondered if Foxa2 would prefer endoderm-specific binding sites in ESCs, where endoderm TFs are not yet expressed.

**Figure 7.**
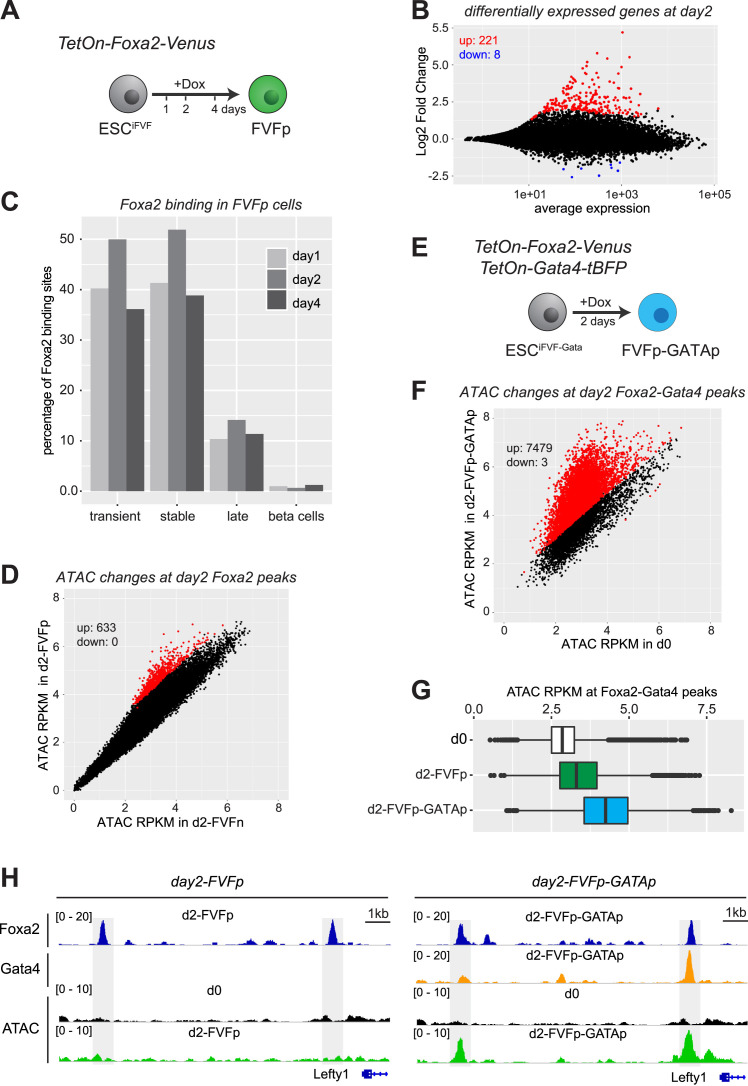
Transcriptional and epigenetic effects of Foxa2 and Gata4 binding in ES cells (**A**) Scheme of the experimental strategy to induce the expression of Foxa2 in ESCs. (**B**) Dot plot showing average expression vs. log2-fold change of protein coding genes in Foxa2-expressing (d2-FVFp) versus non-expressing (d2-FVFn) ESC^iFVF^ cells 48h after dox induction. Genes with significantly changed expression (*P*_adj_ < 0.05, fold change > 2; *n* = 2 for each condition) are coloured (red = increased expression in d2-FVFp, blue = increased expression in d2-FVFn). (**C**) Bar graph showing the percentage of beta cell- or endoderm-specific Foxa2 binding sites bound by Foxa2 in FVFp cells, one, two or four days after dox induction. (**D**) Dot plot showing normalized ATAC-seq coverage in Foxa2-expressing (d2-FVFp) versus non-expressing (d2-FVFn) ESC^iFVF^ cells at Foxa2 binding sites 48 h after dox induction. Significant chromatin accessibility changes (*P*_adj_ < 0.05, fold change > 2; *n* = 2 for each condition) are coloured in red. (**E**) Scheme of the experimental strategy to induce the expression of Foxa2 and Gata4 in ESCs. (**F**) Dot plot showing normalized ATAC-seq coverage in Foxa2/Gata4 co-expressing (d2-FVFp-GATAp) versus non-expressing (d0) cells at Foxa2/Gata4 binding sites 48 h after dox induction. Significant chromatin accessibility changes (*P*_adj_ < 0.05, fold change > 2; *n* = 2 for each condition) are coloured in red. (**G**) Box plots of normalized ATAC-seq coverage of Foxa2/Gata4 co-bound sites in control (d0), Foxa2-expressing (d2-FVFp) and Foxa2/Gata4 co-expressing (d2-FVFp-GATAp) cells. *n* = 2 for each condition. Wilcoxon ranks-sum test statistics is shown in Supplementary Table S7. (**H**) Genome browser view of example Foxa2 binding sites in d2-FVFp cells (right panel, day2-FVFp) and Foxa2/Gata4 co-bound sites in d2-FVFp-GATAp cells (left panel, day2-FVFp-GATAp). The following tracks are displayed: Foxa2 Chip-seq in d2-FVFp and d2-FVFp-GATAp cells; Gata4 ChIP-seq in d2-FVFp-GATAp cells; ATACseq in d0, d2-FVFp and d2-FVFp-GATAp cells. Dashed regions indicate Foxa2 binding sites in d2-FVFp cells (left panel) which are co-bound with Gata4 in d2-FVFp-GATAp cells (right panel).

We engineered doxycycline (Dox) inducible Foxa2-Venus ESCs (ESC^iFVF^), which allowed FACS isolation of Foxa2 expressing ESCs after 1, 2 and 4 days of Dox induction (Figure 7A, Supplementary Figure S7A). To test if Foxa2 expression in ESCs would already be sufficient to activate the endoderm network we performed RNA-seq of Foxa2-expressing (d2-FVFp) versus non-expressing (d2-FVFn) ESC^iFVF^ cells 2 days after Dox induction. We found 229 differentially expressed genes (fold change > 2, *P*_adj_ < 0.05; Figure 7B, Supplementary Table S8). Most genes were upregulated (221 up-regulated, 8 down-regulated), suggesting an activating role of Foxa2. Remarkably, only 72 out of 588 genes which were normally induced during endoderm differentiation were also upregulated in d2-FVFp cells (Supplementary Table S8). Key endoderm TFs were not properly induced (Supplementary Figure S7B), demonstrating that Foxa2 expression in ESCs is insufficient for endoderm differentiation.

Next, we examined to which extent endodermal Foxa2 binding sites are bound by Foxa2 in ESC^iFVF^ cells 1, 2 and 4 days after Dox induction. We found that a large percentage of transient and stable peaks, but less late peaks were bound by Foxa2 in ESC^iFVF^ cells (Figure 7C). In contrast, only ∼1% of pancreas-specific binding sites were bound by Foxa2 in ESCs (Figure 7C). We did not observe striking changes in Foxa2 localization with longer induction times (Figure 7C). These data demonstrate that endodermal Foxa2 binding sites are primed for Foxa2 binding already in ESCs and, that Foxa2 can recognize these sites in the absence of additional endoderm-specific transcription factors. We also conclude that Foxa2 expression alone is insufficient to induce processes which would make pancreas-specific beta cell binding sites accessible.

We then performed ATAC-seq on Foxa2-expressing ESCs (d2-FVFp) to test if Foxa2 can induce chromatin accessibility on its binding sites. Remarkably, only a very small fraction (∼1%) of Foxa2 bound regions displayed significant gains in chromatin accessibility (Figure 7D), suggesting that Foxa2 binding alone is insufficient to trigger chromatin opening. We also performed ChIP-seq analyses for H3K4me1 and H3K27ac in Foxa2-expressing ESCs (d2-FVFp). We detect an increase in H3K4me1 and to lesser extent in H3K27ac (Supplementary Figure S7C,D, Supplementary Table S7). This behaviour mimics our findings for transient vs. stable and late binding sites during endoderm differentiation, where Foxa2 binding coincides with increased active chromatin marks but combinatorial binding with additional TFs was necessary for chromatin opening. We therefore sought for features which could distinguish binding sites showing higher chromatin accessibility vs. binding site which do not change. Firstly, we detected that ATAC-seq coverage was higher in opened Foxa2 binding sites already before Foxa2 induction (Supplementary Figure S7E). Secondly, we found slightly higher enrichment for DNA binding motifs of AP1 family members in opened Foxa2 binding sites (Supplementary Figure S7F). Together, our data are in line with a model in which Foxa2 preferentially binds to primed binding sites without the need for collaborating TFs. Increased chromatin accessibility, however, requires the binding of collaborating TFs in the vicinity.

To examine if collaboration between Foxa2 and additional transcription factors favours chromatin accessibility in the ES cell system, we tested whether co-expression of Foxa2 and Gata4 would result in enhanced chromatin accessibility on co-bound sites. For this experiment we generated an ESC line allowing Dox-mediated induction of both Foxa2 and Gata4 (ESC^iFVF-Gata^, Figure 7E). We isolated Foxa2/Gata4 double-positive cells by FACS sorting (Supplementary Figure S7G) and performed ChIP-seq for Foxa2 and Gata4 as well as ATAC-seq to detect changes in chromatin accessibility. Compared to Foxa2 expressing cells (d2-FVFp), which exhibit marginally increased chromatin accessibility (Figure 7D), Foxa2/Gata4 co-expressing cells (d2-FVFp-GATAp) showed a marked increase in chromatin accessibility on Foxa2/Gata4 co-bound sites (Figure 7F–H). These data, together with our finding that Foxa2 and Gata4 co-binding coincides with increased chromatin accessibility during endoderm differentiation provide strong support for our hypothesis that co-binding of Foxa2 with additional TFs is needed to generate increased chromatin accessibility.

## DISCUSSION

**Figure 8.**
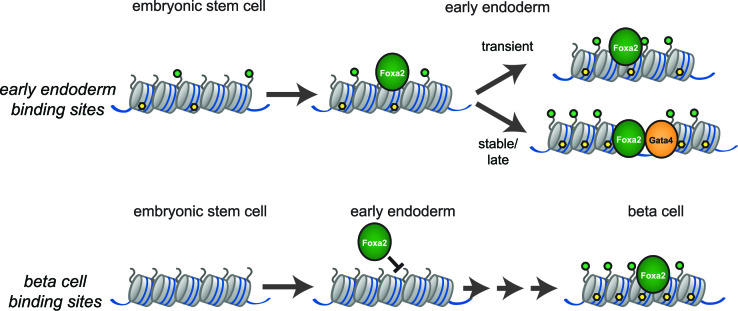
Model for binding site selection and chromatin opening by Foxa2. During the transition from ESC to endoderm, Foxa2 preferentially binds endoderm binding sites featured by low levels of active chromatin modifications (yellow – DNA hydroxymethylation, green – active histone marks). Non-bound lineage-inappropriate binding sites (e.g. beta cell-specific sites) are not featured by active chromatin marks during early endoderm differentiation. Increase in chromatin accessibility occurs on binding sites where Foxa2 co-binds with additional transcription factors (stable and late sites), whereas isolated Foxa2 binding sites do not show increase in chromatin accessibility upon Foxa2 binding (transient sites).

Pioneer transcription factors have critical roles in cell fate specification and are needed for the activation of lineage programs in a cell type-specific manner. How PTFs recognize cell type-specific target sites, and how they initiate remodelling of the surrounding chromatin remains poorly understood. In the present work we addressed these questions by studying the paradigm PTF Foxa2 in the physiological context of endoderm differentiation. Our data support a model by which Foxa2 binding sites are defined by low levels of active chromatin modifications and where local chromatin opening requires co-binding of additional transcription factors in close vicinity (Figure 8). This model is based on the following observations:

- In mouse ESCs, endodermal but not pancreatic Foxa2 binding sites are pre-marked by low levels of active chromatin modifications. This feature is also conserved in human ESCs.
- Foxa2 preferentially binds to endoderm-specific, but not pancreas-specific binding sites when expressed in ESCs.
- During endoderm differentiation, increased chromatin accessibility is observed on binding sites where Foxa2 binds together with other transcription factors, i.e. Gata4.
- In the absence of other endoderm transcription factors, in ESCs, Foxa2 has a very limited activity to induce chromatin opening. In the small subset of binding sites where chromatin accessibility is enhanced, Foxa2 may bind together with additional TFs, e.g. AP1 proteins.
- Co-expression of Foxa2 and Gata4 in ESCs results in enhanced chromatin accessibility at Foxa2/Gata4 co-bound sites.

Our data are in line with recent models in which epigenetic priming determines cellular competence (15,42). In this way the epigenetic landscape of a cell directs transcription factor binding to lineage-appropriate sites. These regions of low level active chromatin modifications are likely to be established by the transcription factor network which is active before lineage decisions are made. In ESCs, pluripotency-associated TFs are likely to be responsible for this epigenetic priming (43).

How epigenetic priming may direct transcription factors binding is still unclear. Pioneer transcription factors, such as Foxa2, can bind specific DNA sequences in the context of nucleosomes. However, these binding sites occur very frequently in the genome. Thus, a limited number of Foxa2 molecules per cell will preferentially enrich on a set of binding sites to which Foxa2 has highest affinity. In the cellular context, affinity is not a function of DNA sequence binding alone, but rather represents a combination of different features including DNA shape, DNA methylation, chromatin organization and protein interactions in the vicinity of the binding site (44). In this context, regions of active chromatin modifications are characterized by higher chromatin dynamics and generally enhanced accessibility, which may favor Foxa2 binding. This is consistent with a recent study (45). Chromatin-modifying enzymes that reside in regions of active chromatin may also target Foxa2, thereby affecting binding affinity. In line with this hypothesis are findings that Foxa2 activity is augmented by p300-mediated acetylation on Lys259 (46), whereas SIRT1-mediated deacetylation leads to reduced Foxa2 stability (47).

From our data it is currently not possible to establish a causative link between a pre-existing chromatin state and Foxa2 recruitment. We attempted to answer this question by targeting H3K27ac to pancreas-specific Foxa2 binding sites using a Cas9-p300 fusion protein (48,49). Unfortunately, we were unable to detect significant levels of H3K27ac (data not shown), suggesting that establishment of a low-level active chromatin state requires more than recruitment of a single chromatin modifying factor.

Another determinant of transcription factor binding site selection could be co-binding with additional transcription factors. For example, sexual dimorphism in liver cancer is determined by differential target activation depending on Foxa1/2 and AR or ERa interactions (50). Similarly, Oct4 occupies different genomic regions when expressed alone or in combination with other reprogramming factors (51). Our data suggest that many Foxa2 binding sites during endoderm differentiation do not require co-binding with additional transcription factors, e.g. transient binding sites. We rather find that co-binding leads to changes in chromatin accessibility, which is likely to be a prerequisite for enhancer activation. We specifically investigated the co-binding of Foxa2 with Gata4 which occurs on stable and late binding sites. However, other endoderm-related transcription factors are likely to act in addition to Gata4 to promote chromatin opening. Interestingly, transient binding sites largely lack binding motifs except for the Foxa2 motif. The function of these binding sites is therefore rather unclear. It is possible that Foxa2 binding on transient sites is important for epigenetic priming of alternative lineages, e.g. distinct cardiac progenitors which derive from the Foxa2^pos^ mesendoderm lineage (52). However, we cannot exclude the possibility that many binding events are neutral and do not have consequences for transcriptional regulation.

How pioneer transcription factors initiate opening of the surrounding chromatin is still poorly understood. Some PTFs recruit chromatin remodeling complexes to alter the local chromatin structure. For example, Oct4 and BZLF1 require Brg1 and INO80 for inducing chromatin changes (53,54). Foxa proteins, in contrast, have been shown to alter chromatin structure in an ATP-independent manner *in vitro* (7), or by displacing the linker histone H1 *in vivo* (17). Interestingly, work from the Kaestner lab (16) demonstrated that Foxa1/2 can also recruit nucleosome remodeling complexes (Nap1l1/SWI/SNF/INO80) which enable nucleosome eviction on their binding sites. Why co-binding of TFs appears necessary for inducing chromatin accessibility is still unclear. As chromatin opening is facilitated by TF-recruited chromatin remodelling activities (55), co-binding of TFs could synergize in recruiting multiple chromatin remodelling machineries. Interestingly, co-binding of transcription factors in close vicinity does not immediately result in enhanced chromatin accessibility. For example, we observed on stable Foxa2 binding sites that chromatin opening is delayed. Although Foxa2 and Gata4 bind already in d3F cells, opening on these binding sites was mainly observed in d5FS cells. Delayed chromatin opening upon PTF binding was also observed recently for the PTF Pax7 in pituitary glands (25). These findings suggest that cell cycle, replication or additional co-factors (56) may be required for inducing higher chromatin accessibility. Experiments that specifically address the combinatorial logic of transcription factor binding and recruitment of chromatin-modifying activities are needed to better understand the requirements for enhancer activation. PTFs are defined by their intrinsic ability to target DNA sites on nucleosomes meaning that their binding should not be affected by nucleosomes. But surprisingly chromatin remodelers can influence the binding of PTFs such as Oct4 and Sox2 (53). This suggests a complex cross talk between remodelers and PTFs that deserves additional studies.

## DATA AVAILABILITY

All genomic data have been deposited in the GEO database under accession number GSE116262. All the genomic data analysed in this study are listed in Supplementary Table S10.

The code underlying our analysis is available upon request.

## Supplementary Material

gkz627_Supplemental_Files
