# Supplementary material for: Pre-marked chromatin and transcription factor co-binding shape the pioneering activity of Foxa2
Source: Nucleic Acids Res. 2019 Jul 27;47(17):9069–86. doi: 10.1093/nar/gkz627 (PMC6753583; doi:10.1093/nar/gkz627)
Supplement: gkz627_Supplemental_Files [file gkz627_supplemental_files.zip › Supplementary material combined.NAR.r1_final.pdf]

Supplementary information for

Pre-marked chromatin and transcription factor co-binding shape the pioneering activity of Foxa2.

List of contents:

Supplementary methods.

Supplementary figures related to: Figure 1, Figure 2, Figure 3, Figure 4, Figure 5, Figure 6, Figure 7.

Supplementary tables:

Table S1_significant_genes_d0_d3F_d5FS.xlsx

Table S2_DARs_gene.association.xlsx

Table S3_motifs_ATAC-DARs.xlsx

Table S4_significant_genes_het_homo.xlsx

Table S5_Foxa2_peaks.xlsx

Table S6_motifs_Foxa2-binding-sites.xlsx

Table S7_wilcoxon.statistics.Boxplots.xlsx

Table S8_Foxa2 expression ESC - regulated_genes.xlsx

Table S9_ATACseq.metrics.xlsx

Table S10_NGS-Sample_list.xlsx

SUPPLEMENTARY METHODS

Generation of Foxa2^{Venus} Knockout cells

Generation of Foxa2^{Venus} knockout targeting vector

The knockout construct was designed as shown in Fig.S3. The pBKS-Venus vector carrying the H2B-Venus sequence (1) was linearized with SalI. To induce a blunt end and a NotI restriction site the 5’ overhangs of the SalI linearized vector were filled up using Klenow enzyme and subsequently cut with NotI. The 3’ homology arm for the Foxa2 gene was cloned from the pBKS-3’HA vector into the linearized pBKS-H2B-Venus vector using the restriction enzymes EcoRV and KpnI, resulting in pBKS-Venus-3’HA. The 5’ homology arm for the Foxa2 gene was amplified from the pL254-Foxa2 vector (2) as a template using primers carrying a NotI site (EP 1447/EP 1448). The amplified 5’ homology arm was cloned via NotI into the pBKS-Venus-3’HA vector, resulting in p-BKS-Venus-3’HA-5’HA vector, containing the H2B-Venus sequence flanked by the 5’ and 3’ homology arms of the Foxa2 gene. In between the H2B-Venus and 3’ homology arm the SV40 polyadenylation signal sequence followed by the loxP-flanked murine PGK and the bacterial EM7 promoter-driven neomycin resistance gene. To ensure the correct insertion of the inserts, the vectors were digested with HindIII/XhoI: pBKS-Venus: 4574bp, 1910bp; pBKS-Venus-3’HA: 4574bp, 1912bp, 484bp; and with ApaLI/XhoI: p-BKS-Venus-3’HA-5’HA: 1246bp, 928bp, 727bp, 602bp wrong orientation: 1246bp, 1206bp, 727bp, 602bp and pBKS-Venus-3’HA: 2601bp, 2396bp, 1246bp, 727bp. Subsequently, the Foxa2-Venus targeting vector was sequenced and the vector with the correct sequence was used for transfection of the mESCs. Prior to transfection of mESCs the targeting vector was linearized using ApaI.

Oligonucleotides used for genotyping and sequencing

EP 1447 NNNGCGGCCGCGGGAATGTGCACTAAAAGGGAGGAAACC

EP 1448 NNNGCGGCCGCGCATACTGGAAGCCGAGTGCATGG

EP 397 CTACTACCAAGGAGTGTACTCC

EP 1520 ACCATTACGCCTTCAACCAC

EP 1513 GGAATTCTGGCCATTCTAGC

EP 1499 GGCTGGACGTAAACTCCTCTTC

Foxa2 3’ Southern probe (732bp):

CTGGATATGCTCTAGAAAGGCAGAAGTTTACAGTTTTTTTAATATCAGGCCTCCTTTCTAGTCAGTGA
ACTTAGACTGGGTTTACCAATTTTGGTGCATGGCTCTTCCAGCTACTTGAAGCATTGCCCCCCCTAGA
CCTTCCTGTGCCATTGAGACTACCTGGCTCTAGGTTGTGCCGGGAGGGCAGCCTGTCTCAGTCTCAC
AGGTGTTATCCAGGTATTGGGAAACCTTGCTAGGCTAGGAACGATGAGCCACCTAATCTGGGGAAA
CATTTTAACATTGGGAATTGGGTATAATTGCATAGTTAAGGGTAACCCCCAAATCTTTTATTAAGAAG
TTATTCTGTGGGTGGGGAGATAGGGAGGGATGGAAGGGTGCCCTGAGCAGCTTAGCAAATGACTC
CCAAAGTAGTGAAATCCCAGTGTCTCAGGAATGGTGTCTCCCTTCTACCAGCCAGGGCAAAGCTGTT
TGTTAGCTTAGGAAGCTCCTATAGGCAAACCACACTTGAGGCCCAGGGACTGAATGGGTATTTTGTG
AGCCTCCAGGAAAATACAAAGACCCCAAATAAAACCTCACCAATCATTTCCACCACTCTGCAGATTTT
CCAAATTGACGGGTAACTGTAGAGGAGGTCGTGTTTTGCAAAAGGAGCCTCCTCACGCTGACCTGC
ATCTCCTGCCCTTGAAGCTGTCCCTCCCGCCCGCCCCCAGTCTGACTTTCCATAGGCCATTC

Design of guide RNAs (sgRNAs)

To design the gRNAs the online tool Optimized CRISPR Design (crispr.mit.edu/) was used. 250 nucleotides of the coding region in exon 3 were submitted as template for the gRNA design. The selected gRNA pair had a 52 bp 5’ overhang and 17 bp gRNA offset.

The submitted sequence to Optimized CRISPR Design:

CTCTATCAACAACCTCATGTCGTCCGAGCAGCAACATCACCACAGCCACCACCACCATCAGCCCCACA
AAATGGACCTCAAGGCCTACGAACAGGTCATGCACTACCCAGGGGGCTATGGTTCCCCCATGCCAG
GCAGCTTGGCCATGGGCCCAGTCACGAACAAAGCGGGCCTGGATGCCTCGCCCCTGGCTGCAGACA
CTTCCTACTACCAAGGAGTGTACTCCAGGCCTATTATGAACTCATCCTAA

The selected gRNAs:

gRNA #4 forward 5‘-3‘: CACCGGGGGATGAGTTCATAATAGGCC

gRNA#4 reverse 3‘-5‘: CCCCCTACTCAAGTATTATCCGGCAAA

gRNA#12 forward 5‘-3‘: CACCGGGGCCCTGCTAGCTCTGGTCAC

gRNA#12 reverse 3‘-5‘: CCCCGGGACGATCGAGACCAGTGCAAA

Cell Culture and genome targeting of mESCs via CRISPR-Cas9D10a

Mouse embryonic stem cells (mESCs) were cultured on MMC-treated murine embryonic feeders (MEFs) in Dulbecco’s Modified Eagle Medium (DMEM, Invitrogen) containing 15% fetal calf serum (FCS, PAN), 2 mM L-glutamine (Invitro-gen), 100 lM b-mercaptoethanol (Invitrogen) and 1,500U/ml LIF (self-made).
Prior to electroporation, the Foxa2-Venus targeting vector was linearized via ApaI and subsequently together with the gRNAs and Cas9D10A cleaned up by phenol-chloroform extraction followed by EtOH precipitation. For each electroporation half a 10 cm dish of confluent IDG3.2 mESCs (3) was used. The medium was refreshed the next day. After 24 hours the selection of the cells was initiated with 300 µg/ml geneticin (G418).

Generation of Doxycycline-inducible Foxa2-Venus ES cells

We made use of a lentiviral Tet-On-3G system that consists of two lentiviral vectors: a regulator vector that stably expresses the Tet-On 3G transactivator protein (1702_pLenti6-EF1a-Tet3G-IRES-Neo), and a response vector that contains the TRE3G promoter that controls the expression of Foxa2-Venus (1701_pLenti6-TRE3G-Foxa2-Venus-PGK-PuroR).

For the 1702_pLenti6-EF1a-Tet3G-IRES-Neo, a Human EF1a Promoter, a Tet-On 3G element and a Neo resistance gene were cloned into pLenti6-puro backbone (4) using Gibson Assembly as described (5). The Human EF1a Promoter/Tet-On 3G fragment as well as the Neo(R) cDNA were PCR amplified from the pEF1α-Tet3G (Cat. # 631336, Clontech) using the following primer pairs

F: gggacagcagagatccactttggccgcggctcgaggagtaattcatacaaaaggactcg,

R: ccgatcgatagatcttcatgtctggatccttacttagttacc

and

F: tgccttgtaagtcattggtcttaaaggtaccctcagaagaactcgtcaagaag,

R: accgggccggatatcatgattgaacaagatggattgc, respectively. The IRES2 fragment was PCR amplified from the pTRE3G-IRES (Cat. # 631174, Clontech) using the primer pairs

F: aggatccagacatgaagatctatcgatcggccg,

R: atcttgttcaatcatgatatccggcccgg. T

he pLenti6 backbone was cut with XhoI and KpnI.

The 1701_pLenti6-TRE3G-Foxa2-Venus-PGK-PuroR was cloned in two steps using Gibson Assembly. First, the Foxa2 cDNA was amplified from the pHD-Foxa2 vector (Kindly provided by Klaus Kaestner) using the following primer pairs

F:tttccgtaccacttcctaccctcgtaaagtcgacaccggggcccagatctATGCTGGGAGCCGTGAAGATGGAAGGG

R: GGTGAACAGCTCCTCGCCCTTGCTCACCATTCTAGAGGATGAGTTCATAATAGGCCTGGAGT

and the Venus cDNA was amplified using the following primer pairs

F: GAGTGTACTCCAGGCCTATTATGAACTCATCCTCTAGAATGGTGAGCAAGGGCGAGGAGCTG,

R: agaatttcgtcatcgctgaatacagttacattggatccctgcaggctagcTTACTTGTACAGCTCGTCCATGCC.

Both fragments were then assembled together with a pTRE3G-IRES (Cat. # 631174, Clontech) backbone, cut with BglII and NheI. From the resulting vector, the TRE3G-Foxa2-Venus cassette was amplified using the following primer pair

F: tttattacagggacagcagagatccactttggccgcggctaggcgtatcacgaggccctt,

R: ctgccttggaaaaggcgcaaccccaaccccggatccctgcaggctagc and assembled together with a pLenti6-puro backbone, cut with XhoI.

Lentiviral particles were generated using standard protocols. After transduction SCF-ES cells (Sox17-Cherry homozygous, derived from the same batch of blastocysts as DKI cells) were selected with Puromycin (1ug/ml) and Neomycin (250ug/ul). Foxa2 expression was induced in cell lines T119 and T128 with 40ng/ul doxycycline for 1 day, 2 days or 4 days followed by FACS isolation. Cells were routinely tested for mycoplasma contamination.

Generation of Doxycycline-inducible Foxa2-Venus, Gata4-tagBFP ES cells

We made use of the Tet-On-3G system already present in the Doxycycline-inducible Foxa2-Venus ES cells (cell line T128).

A lentiviral vector containing the TRE3G promoter controlling the expression of Gata4 (1722_pLV[Tet]-Bsd-TRE3G-mGata4-T2A-TagBFP) was generated by VectorBuilder (Vector ID is VB180822-1113dwn which can be used to retrieve detailed information about the vector on vectorbuilder.com).

Lentiviral particles were generated using standard protocols. After transduction the T128 cells were selected with Blasticidin (5ug/ml). Foxa2 and Gata4 expression were induced in cell line T134 with 40ng/ul doxycycline for 2 days followed by FACS isolation. Cells were routinely tested for mycoplasma contamination.

Immunohistochemistry

The Foxa2^{Venus} mESCs were differentiated under endoderm conditions on IbiTreat µ-Slide 8 well chambers for 3 days. The mESCs were fixed in 4% PFA in DPBS for 5 min at RT, washed once and permeabilized for 10 min at RT using 0.1 M glycine and 0.1 % triton X - 100 in MilliQ water. Followed permeabilization, the cells were rinsed 2 x with DPBST and blocked in DPBST containing 0.1 % Tween - 20, 10 % heat inactivated fetal calf serum (FCS), 0.1 % BSA and 3 % donkey serum for 30 minutes at 37°C. Subsequently, the blocking solution was replaced by primary antibodies diluted in blocking solution: α-Foxa2 1:1000 (Cell signaling #8186) and α-GFP 1:1000 (Aves Labs #1020), α- Oct4 1:500 (Santa Cruz #5279), α-Sox17 1:500 (Acris/Novus #GT15094), α-T 1:300 (Santa Cruz #17743), α-Cer1 1:500 (R&D #AF1075) and incubated for 3 hours at RT while shaking. The cells were washed three times for 5 minutes each and afterwards incubated for 1 hour at RT in blocking solution containing the following secondary antibodies (1:800): α- chicken (Dianova #703-225-155) and α-rabbit (Invitrogen #A21206), α-mouse (Invitrogen #A31570), α-goat (Invitrogen #A21432). The cells were stained with DAPI 1:500 and washed three times with DPBST before taking pictures with a Leica SP5 confocal microscope.

Quantification and statistical analysis

Statistical analysis

Statistical analysis of all bar plots was performed using the Wilcoxon rank-sum test and is shown in Table S7.

RNA-seq

Paired end or single end reads were aligned to the mouse genome version mm10 using STAR (6) with default options "--runThreadN 32 --quantMode TranscriptomeSAM GeneCounts --outSAMtype BAM SortedByCoordinate". Read counts for all genes were normalized using DESeq2 (7). Significantly changed genes were determined through pairwise comparisons using the DESeq2 results function (log_2 fold change threshold=1, adjusted p-value <0.05). For the endoderm differentiation time course analysis, pairwise comparisons between all states (d0, d3F, d5FS) were performed with Deseq2 to isolate a list of differentially expressed genes during endoderm differentiation. Heatmap with differentially expressed genes was plotted with pheatmap using rlog-normalized expression values. PCA analyses were done using the plotPCA function of the DESeq2 package. Bargraphs showing expression data for selected genes were plotted using ggplot2 with RSEM-normalized data (TPM = Transcript Per Million).

ATAC-seq

ATAC-seq reads were aligned to the mouse genome mm10 using Bowtie (8) with options "-q -n 2 --best --chunkmbs 2000 -p 32 -S". ATAC peaks over Input background were identified using Homer (9) findPeaks.pl with option "-style factor". Peaks from all samples were merged using mergePeaks resulting in a unified Peak set. The peak list was filtered for promoter-associate peaks (distance to TSS < 1000bp) with bedtools. Raw ATAC coverage counts were then calculated with annotatePeaks within 500bp across the peak centers. Differential ATAC peaks were determined with the DESeq2 result function and filtered for adjusted p-value < 0.05 and log2 fold change > 1. Genomic feature annotation of ATAC-seq peaks was done using annotatePeaks. Transcription factor motif prediction was done with findMotifsGenome.

ATAC coverage on Foxa2 peaks was calculated using homer annotatePeaks from replicate experiments.

ATAC-seq quality control was performed by investigating the nucleosomal pattern of the bioanalyzer profile of the ATAC-seq libraries, the number of mapped reads and the fraction of reads in peaks vs mapped reads (Supplementary Table S9).

ChIP-seq

ChIP-seq reads were aligned to the mouse genome mm10 using Bowtie with options "-q -n 2 --best --chunkmbs 2000 -p 32 -S". Transcription factor peaks vs Input background were identified using findPeaks with option "-style factor". To define specific peaks for d3F and d5FS stages, Foxa2 peaks which were shared between replicates and showed strong enrichment over input (fc>10) were identified and sorted into d3F, d3F+5FS and d5FS using the homer mergePeaks tool.

For the overlap analysis of Foxa2 binding in ES cells, endoderm and beta cells, high-confidence binding sites were defined with fold change over input > 5 and stage-specific binding sites were counter selected with fold change over input < 2.

Annotation of genomic regions was based on homer annotatePeaks. Transcription factor motif analysis was done with findMotifsGenome. GREAT analysis was done using the online resource at http://great.stanford.edu/public/html.

Coverage of histone modifications was calculated with annotatePeaks from corresponding Tag Directories. Replicate experiments were merged. Boxplots were generated with ggplot2.

Density plots for histone modifications were based on high confidence non-promoter peaks of Foxa2 in d3F and beta cells, as well as Nanog and Trim28 binding sites. Coverage density was calculated with annotatePeaks using option "-size 5000 -hist 50" from corresponding tag directories. Replicate experiments were averaged.

TF binding site predictions

TF binding site plots across Foxa2 binding sites were generated using annotatePeaks with option -m to detect TF motif occurrence within 5000bp of each Foxa2 binding site. Density plots were then generated using ggplot2.

Ranking of transcription factors.

Putative lead TFs were assessed by adopting a previously published approach (10). For each transcription factor we established a sphere of influence of up to three level depth using gene-gene relations based on the STRING database version 10.5 (11). We considered only relations with total scores >300, where less than half of the value was attributed to text mining. Using the measured difference in RNA-seq expression we calculated scores, one for the TF and one for its underling network using the following equations:

$$Score_{TF} = log(FC_{TF})(-\log(AdjPval_{TF}))$$

$$Score_{Network} = \sum_{g=1}^{n} \frac{|log(FC_g)|(-\log(AdjPval_g))}{Dist_g \ Pnd_g}$$

where: TF – transcription factor, FC – fold change, AdjPval – adjusted p-value (both from DSeq2), g – gene, Dist – number of steps between g and TF, Pnd – parent node degree, n - length of list of genes associated with TF.

Subsequently all factors were ranked based on combined ranking of both scores and TFs with expression values below 2 TPM at posterior stage were removed. The TFs were then plotted as network using cytoscape 3.6 (12) with edges width correlating to the STRING interaction score. Only factors connected to other TFs were plotted and the top 5 ranked TFs were highlighted by increase node size.

Ontology Annotation

In silico functional annotation of different groups of Foxa2 binding sites and dynamic ATAC regions were performed with the GREAT tool (13), using the default settings and the whole mouse genome as background. The terms belonging to various gene annotations (GO Biological Process, MSigDB Pathway) were considered. Differentially regulated genes defined by DESeq2 were assigned to GO biological process groups (Annotation data set: GO biological process complete release 20181115) using the PANTHER Overrepresentation Test (release 20181113) (14) with default parameters and with all mouse genes as background.

SUPPLEMENTARY FIGURES

Supplement related to Figure 1

A) Representative FACS plots for pluripotent and differentiated ESCs at day0, day3 and day5 of *in vitro* endoderm differentiation. Differentiated cells were sorted based on expression of Foxa2 and Sox17.

B) Principal component analysis of gene expression profiles determined by RNA-seq from FACS sorted d0, d3F and d5FS cells. Dots of the same colour represent biological replicates.

C) Gene Ontology analysis showing selected biological processes enriched in genes differentially expressed in the transitions d0-d3F and d0-d5FS.

Supplement related to Figure 2

A) Percentages of all ATAC-seq peaks associated with different genomic features in FACS sorted d0, d3F and d5FS cells.

B) Table showing the percentages of differentially expressed genes (UP and DOWN in the indicated comparison) linked to dynamic accessible regions.

C) Principal component analysis of all ATAC-seq peaks, promoter ATAC peaks or non-promoter ATAC peaks detected in FACS sorted d0, d3F and d5FS cells. Dots of the same colour represent biological replicates.

D) Gene ontology analysis showing enriched biological processes at differentially accessible regions d0 vs. d3F.

E) Gene ontology analysis showing enriched biological processes at differentially accessible regions d0 vs. d5FS.

F) Gene ontology analysis showing enriched biological processes at differentially accessible regions d3F vs. d5FS.

Supplement related to Figure 3

A) Targeting strategy for the Foxa2H2B-V allele. The whole open reading frame of Foxa2 was replaced by the H2B-Venus sequence followed by the SV40 poly-A signal sequence and the loxP-flanked murine PGK promoter-driven neomycin resistance gene. The construct is flanked by the 3' and 5' homology arms for Foxa2. The 3' and 5' UTRs of Foxa2 are illustrated by black boxes, the predicted promoter regions by orange boxes and the coding region by red boxes. The Cas9D10A cleaving site is depicted as a red arrowhead. Primer binding sites are indicated by horizontal arrows and primers were used for genotyping PCR (EP 397, EP 1499, EP 1513) and indel PCR (EP 1520, EP 1513). The location of the 3' and 5' southern probes and the restriction sites are shown.

B) The mESC clones were genotyped with the primers EP 1513, EP 397 for the WT allele (649bp) and EP 1513, EP 1499 for the targeted allele (740bp). Out of 24 clones 3 clones were homozygous (12,5%), 7 clones were heterozygous (29%) and 14 clones were WT (58,5%). Indel mutations were detected by the primers EP 1513 and EP 1520 flanking the gRNA binding sites resulting in an 878bp DNA amplicon, which was sequenced. Representative sequences of the Foxa2 locus targeted by Cas9D10A are shown with gRNAs 4/12 target sites and PAMs are indicated by blue and red font color, respectively. The Cas9D10A cleaving site is illustrated by a red arrowhead. Representative indels are shown in selected sequences. Out of 7 heterozygous clones 2 clones showed the WT sequence (WT, 28,6%), whereas 4 clones had deletions (Del, 57%) and one clone had insertions (Ins, 14,4%).

C) Southern blot of mESCs digested with HindIII and hybridized with the 3' southern probe showing the 20012bp WT allele and 6800bp targeted allele. The first Foxa2 (V/V) clone shows residual WT allele from the MEFs used for mESC maintenance.

D) Confocal sections showing differentiated Foxa2 (V/V) and Foxa2 (V/+) mESCs at day 3 of endoderm differentiation stained with antibodies against GFP (green), DAPI (blue), Foxa2 (red). Scale bar: 10µm. Del: deletion, Ins: insertion, MEFs: mouse embryonic fibroblasts.

E) Representative FACS plots for Foxa2$^{Venus/+}$ undifferentiated cells (d0^{con}), Foxa2$^{Venus/+}$ day3 (d3^{con}) and Foxa2$^{Venus/Venus}$ day3 (d3^{KO}) endoderm differentiating cells. The gates indicate Venus positive endoderm differentiating cells.

F) Principal component analysis of gene expression profiles obtained by RNA-seq from FACS sorted pluripotent control ($d0^{con}$) and Foxa2 ko ($d0^{ko}$) cells and endoderm differentiating control ($d3^{con}$) and Foxa2 ko ($d3^{ko}$) cells. Dots of the same colour represent biological replicates.

G) Gene Ontology analysis showing the top 10 (ranked by their p-value) enriched biological processes in genes downregulated or upregulated in endoderm differentiating Foxa2 ko cells ($Foxa2^{Venus/Venus}$).

H) Average expression levels of pluripotency markers detected by RNA-seq in pluripotent control ($d0^{con}$) and endoderm differentiating control ($d3^{con}$) and Foxa2 ko ($d3^{ko}$) cells. TPM: Transcripts Per Kilobase Million. Error bars depict standard deviation (n=2 for d0, n=3 for d3).

I) Average expression levels of mesoderm markers detected by RNA-seq in pluripotent control ($d0^{con}$) and endoderm differentiating control ($d3^{con}$) and Foxa2 ko ($d3^{ko}$) cells. TPM: Transcripts Per Kilobase Million. Error bars depict standard deviation (n=2 for d0, n=3 for d3).

J) Average expression levels of endoderm markers detected by RNA-seq in day0 control ($d0^{con}$), day3 control ($d3^{con}$) and day3 ko ($d3^{ko}$) cells. TPM: Transcripts Per Kilobase Million. Error bars depict standard deviation (n=2 for d0, n=3 for d3).

Supplement related to Figure 4

A) Motif analysis in Foxa2 binding sites. Homer findMotifsGenome was used to identify enriched transcription factor binding motifs within 200 bp of Foxa2 peak summits. The top-scoring motif was in all cases representative of the Foxa family.

B) Gene ontology analysis for biological processes enriched at transient, stable and late Foxa2 binding sites.

C) Enrichment analysis for Molecular Signatures Database (MSigDB) pathways at transient, stable and late Foxa2 binding sites. Red and blue arrows indicate Wnt-signaling and Foxa network related pathways, respectively.

D) Bar plot showing the fraction of differentially expressed genes in d0 vs. d5FS cells bound by Foxa2.

E) Bar plot showing the fraction of differentially expressed genes in control vs. Foxa2 ko cells bound by Foxa2.

F) Scatter plots showing normalized ATAC-seq signals at Foxa2 transient binding sites. From left to right: ATAC-seq signal in d0 vs. d3F, d0 vs. d5FS, control vs. Foxa2 ko cells. Significant chromatin accessibility changes (padj < 0.05, log2 fold change > 1) are coloured in red.

G) Scatter plots showing normalized ATAC-seq signals at Foxa2 stable binding sites. From left to right: ATAC-seq signal in d0 vs. d3F, d0 vs. d5FS, control vs. Foxa2 ko cells. Significant chromatin accessibility changes (padj < 0.05, log2 fold change > 1) are coloured in red.

H) Scatter plots showing normalized ATAC-seq signals at Foxa2 late binding sites. From left to right: ATAC-seq signal in d0 vs. d3F, d0 vs. d5FS, control vs. Foxa2 ko cells. Significant chromatin accessibility changes (padj < 0.05, log2 fold change > 1) are coloured in red.

I) Genome browser view of example transient, stable and late Foxa2 binding sites. The following tracks are displayed: Foxa2 ChIP-seq in d3F and d5FS cells; ATAC-seq in d0, d3F, d5FS, Foxa2$^{Venus/+}$ (con) and Foxa2$^{Venus/Venus}$ (ko) endoderm differentiating cells. Dashed regions indicate Foxa2 binding sites.

J) Genome browser view of example transient, stable and late Foxa2 binding sites. The following tracks are displayed: Foxa2 ChIP-seq in d3F and d5FS cells; ATAC-seq in d0, d3F, d5FS cells; H3K27ac ChIP-seq in d0, d3F, d5FS cells; H3K4me1 ChIP-seq in d0, d3F, d5FS cells. Dashed regions indicate Foxa2 binding sites.

Supplement related to Figure 5

A) Genome browser view of example stable and late Foxa2 binding sites. The following tracks are displayed: Foxa2 ChIP-seq and Gata4 ChIP-seq in d3F and d5FS cells; ATAC-seq, H3K27ac ChIP-seq and H3K4me1 ChIP-seq in d0, d3F, d5FS cells. Dashed regions indicate Foxa2 binding sites.

B) Scatter plots showing normalized ATAC-seq signals in d0 vs. d5FS cells at Foxa2/Gata4 co-bound sites. Significant chromatin accessibility changes (padj < 0.05, log2 fold change > 1) are coloured in red.

Supplement related to Figure 6

A-C) Genome browser view showing Foxa2 ChIP-seq tracks in endoderm and beta cells, Nanog ChIP-seq in day0 cells and indicated chromatin modifications in d0 cells. Dashed areas show a Nanog binding site in day0 cells (A), a Foxa2 binding site in endoderm (B), a Foxa2 binding site in beta-cells (C). Green and red indicate active and repressive chromatin marks, respectively.

D) Read-density heat map showing the normalized coverage of the following features at Foxa2 transient binding sites (from top): Foxa2 ChIP-seq in d3F, d5FS and beta cells; H3K4me1, H3K27ac, H3K4me3 ChIP-seq and 5hmC meDIP-seq in d0 cells.

E) Read-density heat map showing the normalized coverage of the following features at Foxa2 stable binding sites (from top): Foxa2 ChIP-seq in d3F, d5FS and beta cells; H3K4me1, H3K27ac, H3K4me3 ChIP-seq and 5hmC meDIP-seq in d0 cells .

F) Read-density heat map showing the normalized coverage of the following features at Foxa2 late binding sites (from top): Foxa2 ChIP-seq in d3F, d5FS and beta cells; H3K4me1, H3K27ac, H3K4me3 ChIP-seq and 5hmC meDIP-seq in d0 cells .

G) Read-density heat map showing the normalized coverage of the following features at Foxa2 beta cell binding sites (from top): Foxa2 ChIP-seq in d3F, d5FS and beta cells; H3K4me1, H3K27ac, H3K4me3 ChIP-seq and 5hmC meDIP-seq in d0 cells .

H) Box plot showing the normalized coverage of the indicated chromatin modifications at different categories of Foxa2 binding sites in d0 cells. Transient, stable and late binding sites feature higher levels of active modifications. Wilcoxon ranks-sum test statistics is shown in Table S7.

I) Box plots showing normalized ChIP-seq coverage of active (H3K4me1, H3K27ac, H3K4me3) and repressive (H3K27me3) chromatin marks for endoderm vs. liver FOXA2 binding sites in human ESCs. Endoderm binding sites feature higher levels of active modifications. Wilcoxon ranks-sum test statistics is shown in Table S7.

Supplement related to Figure 7

A) Representative FACS plots of ES^{iFVF} cells in non-induced (no doxycycline) and induced (plus doxycycline) conditions.

B) Average expression levels of endoderm marker genes detected by RNA-seq in d0 cells, d5FS cells and d2-FVFp cells. TPM: Transcripts Per Kilobase Million. Error bars depict standard deviation (n=2).

C) Box plot showing normalized H3K4me1 coverage in d0 and d2-FVFp cells at Foxa2 binding sites in d2-FVFp cells. Wilcoxon ranks-sum test statistics is shown in Table S7.

D) Box plot showing normalized H3K27ac coverage in d0 and d2-FVFp cells at Foxa2 binding sites in d2-FVFp cells. Wilcoxon ranks-sum test statistics is shown in Table S7.

E) Box plot showing normalized ATAC-seq coverage of d2-FVFp Foxa2 binding sites in do ESCs (ATAC UP – increased chromatin accessibility in d2-FVFp cells; ATAC NC – no change in chromatin accessibility in d2-FVFp cells). Wilcoxon ranks-sum test statistics is shown in Table S7.

F) Jun-AP1 motif density at d2-FVFp Foxa2 binding sites with (ATAC UP) or without (ATAC NC) changes in chromatin accessibility.

G) Representative FACS plots of $ES^{iFVF\text{-}Gata}$ cells in non-induced (no doxycycline) and induced (plus doxycycline) conditions.

Supplement related to Figure 1

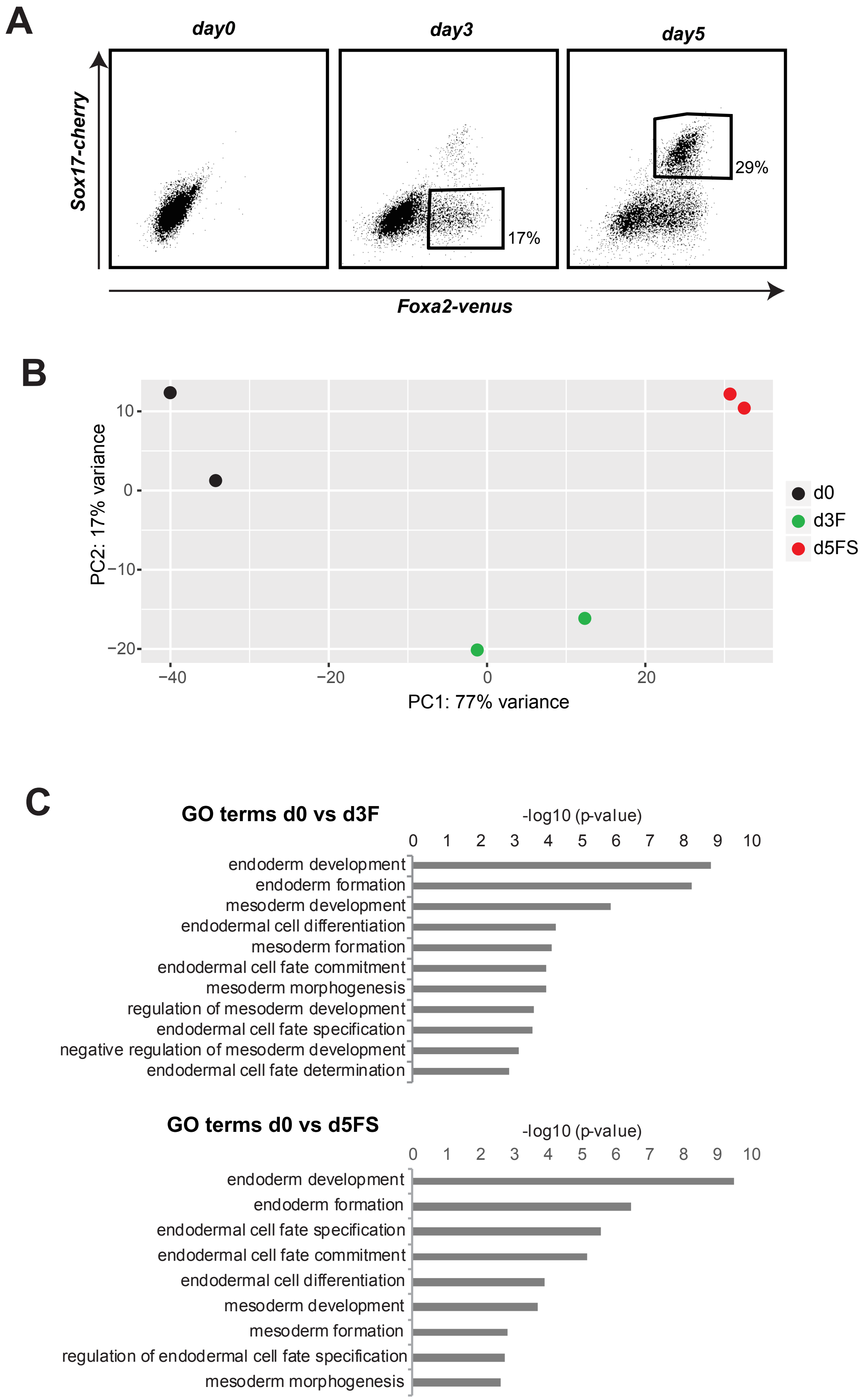

Supplement related to Figure2

A

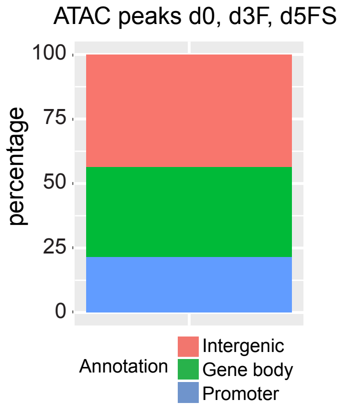

B

Gene expression and ATAC changes

transition	% genes UP	% genes DOWN
d0-d3F	19	46
d3F-d5FS	21	32
d0-d5FS	45	78

C

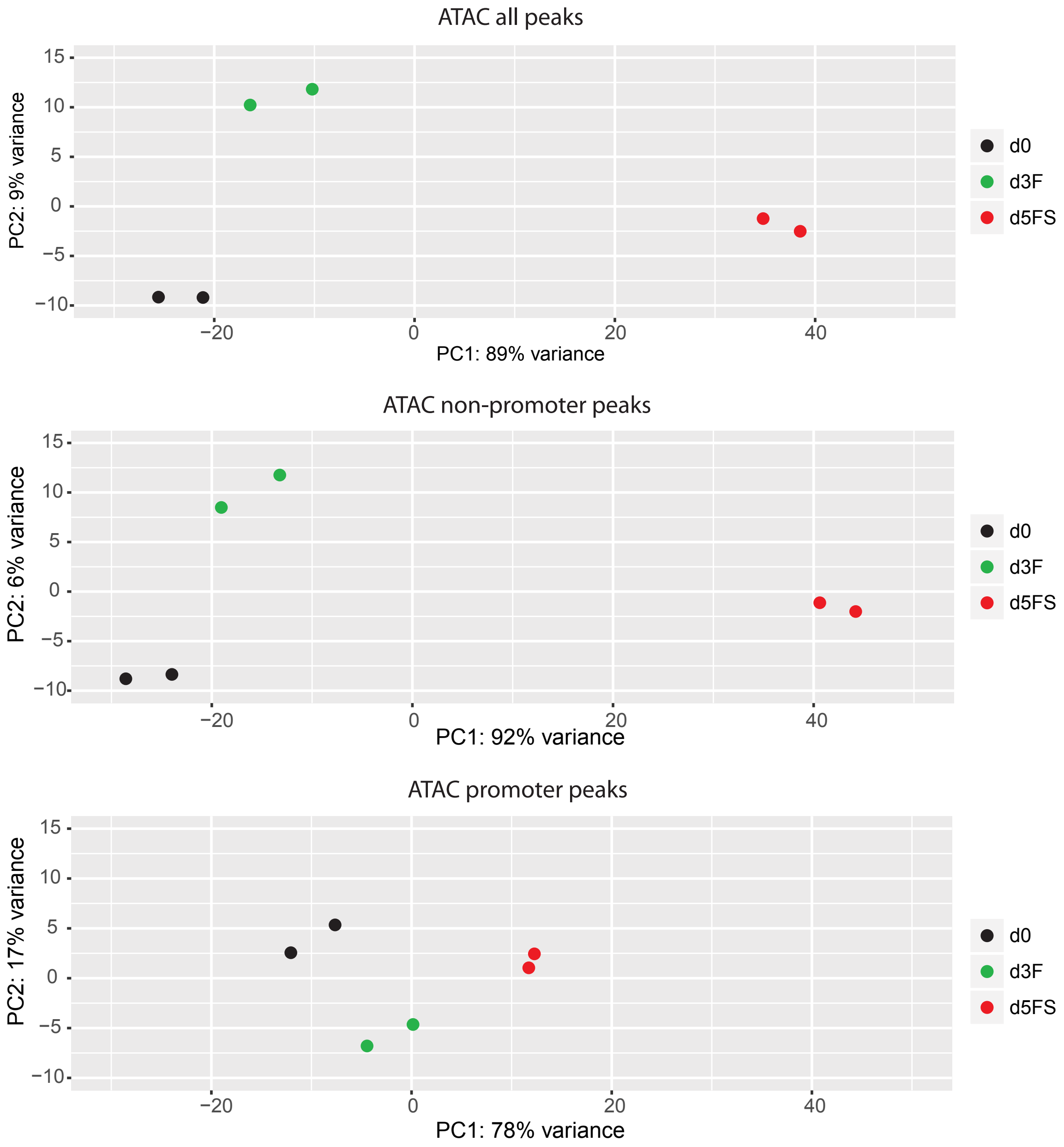

Supplement related to Figure2

D

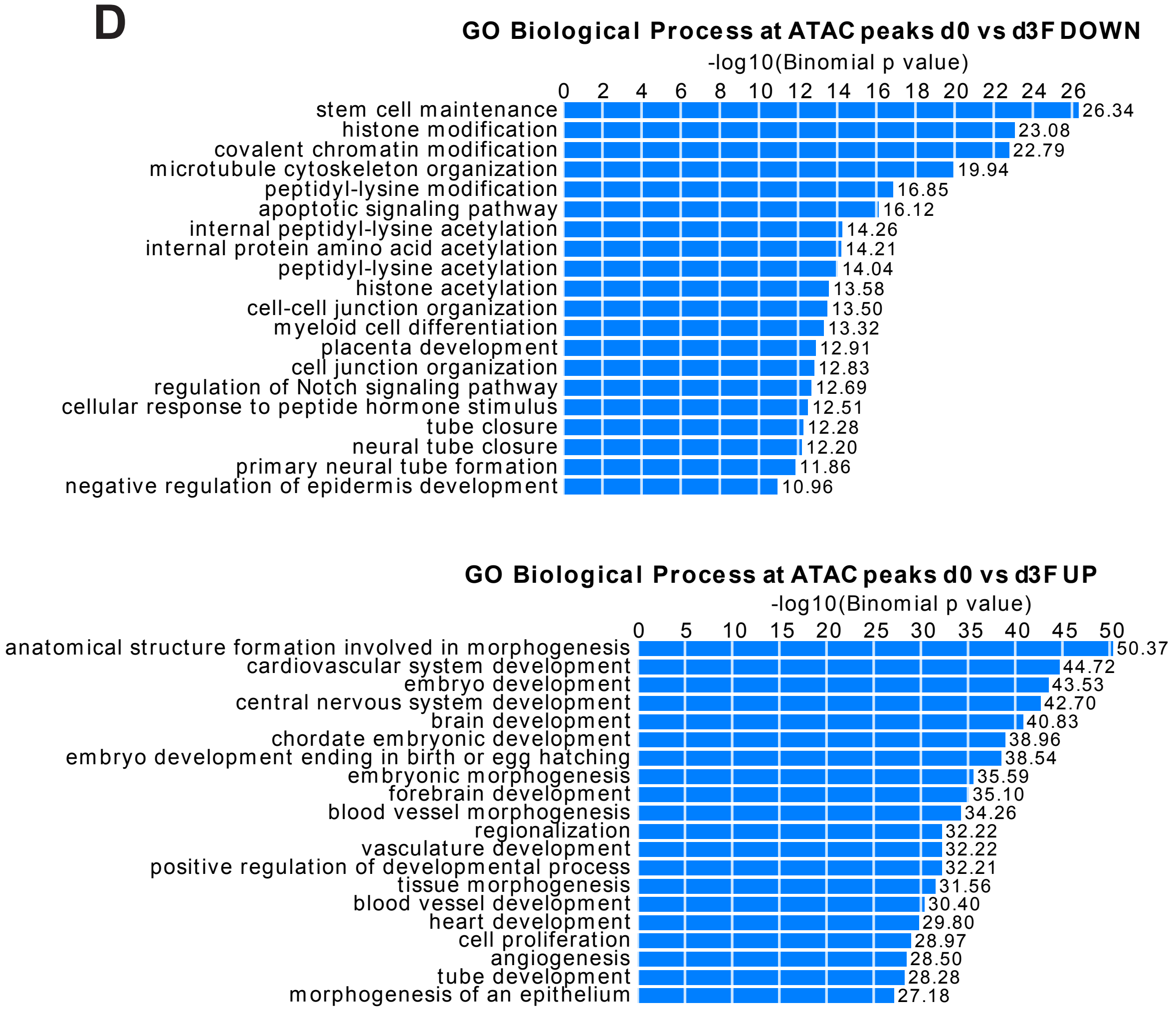

E

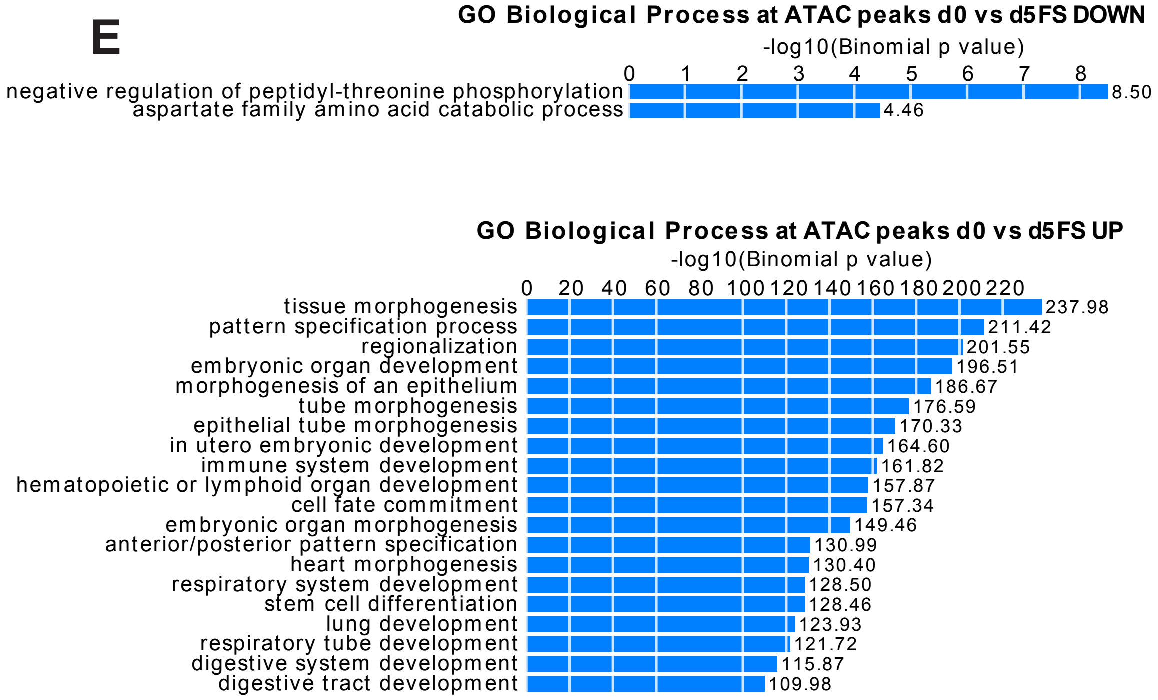

Supplement related to Figure2

F

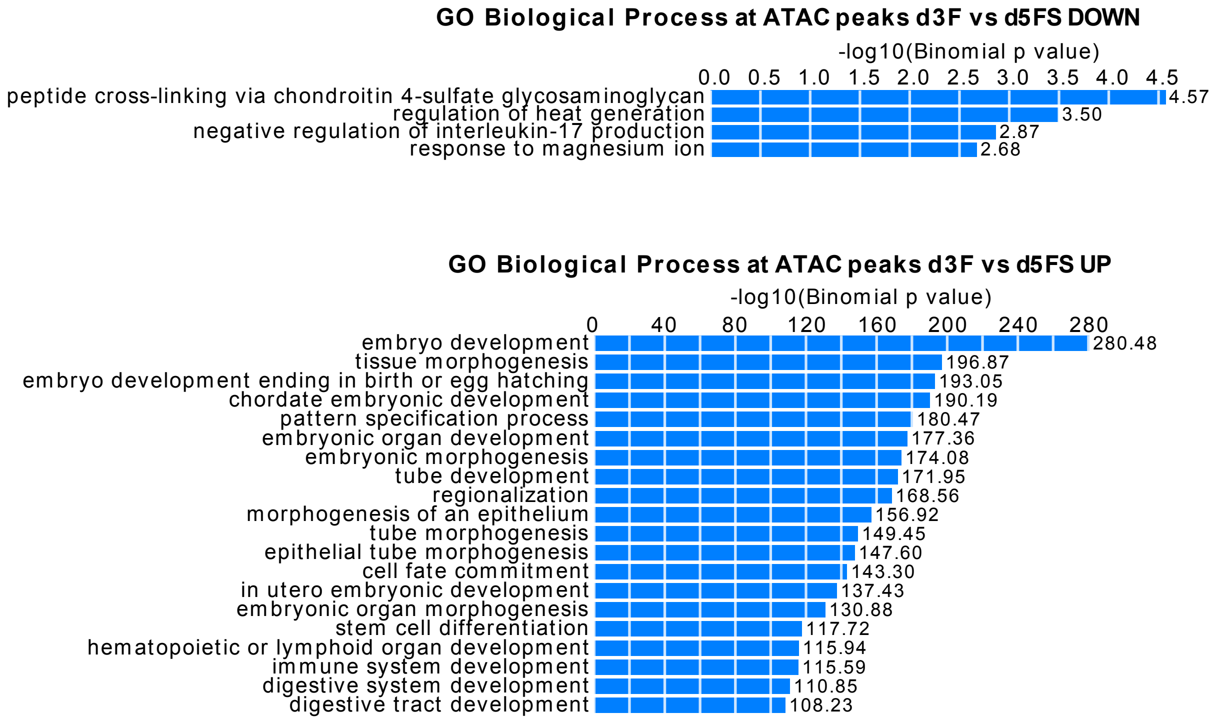

Supplement related to Figure 3

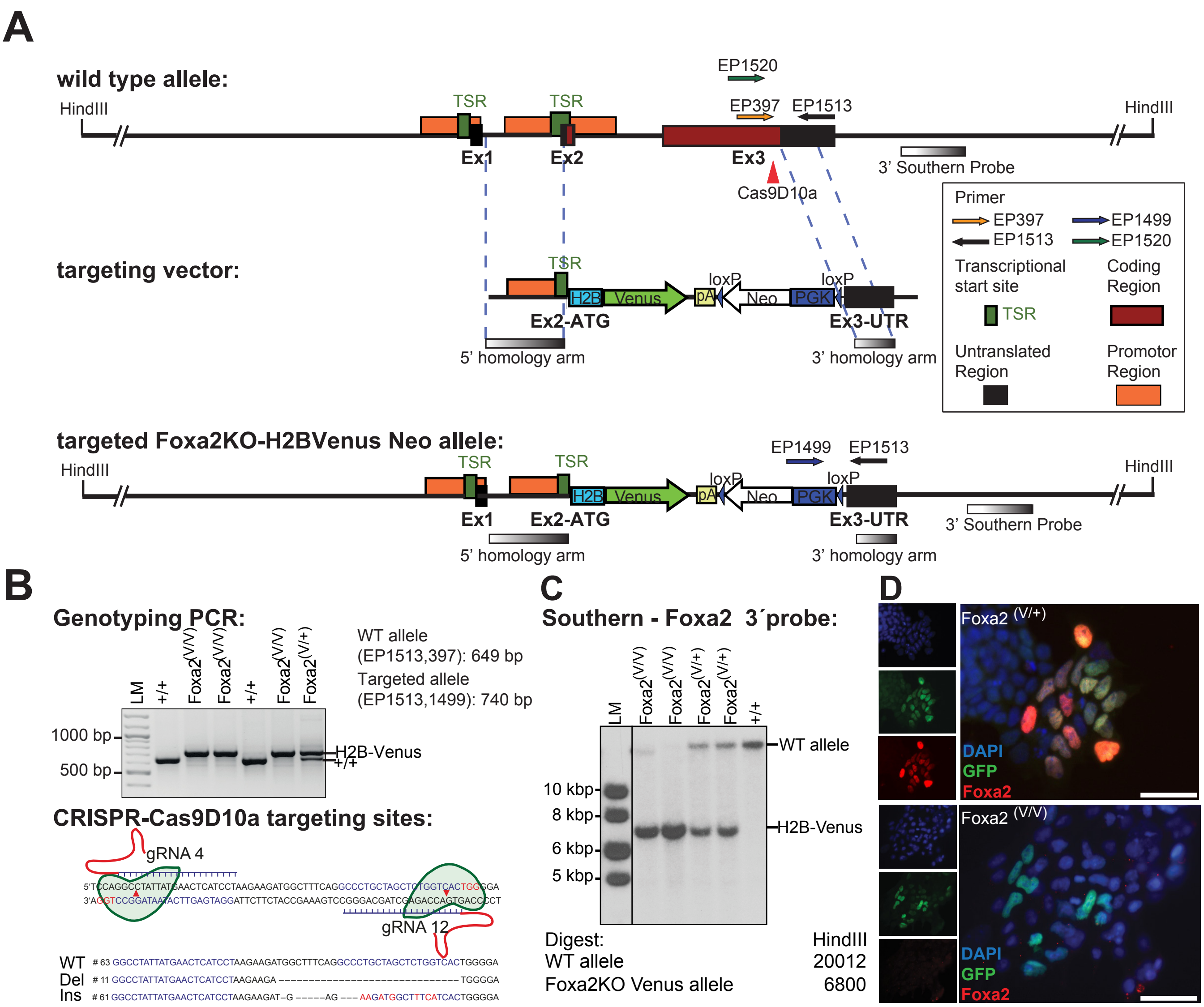

Supplement related to Figure3

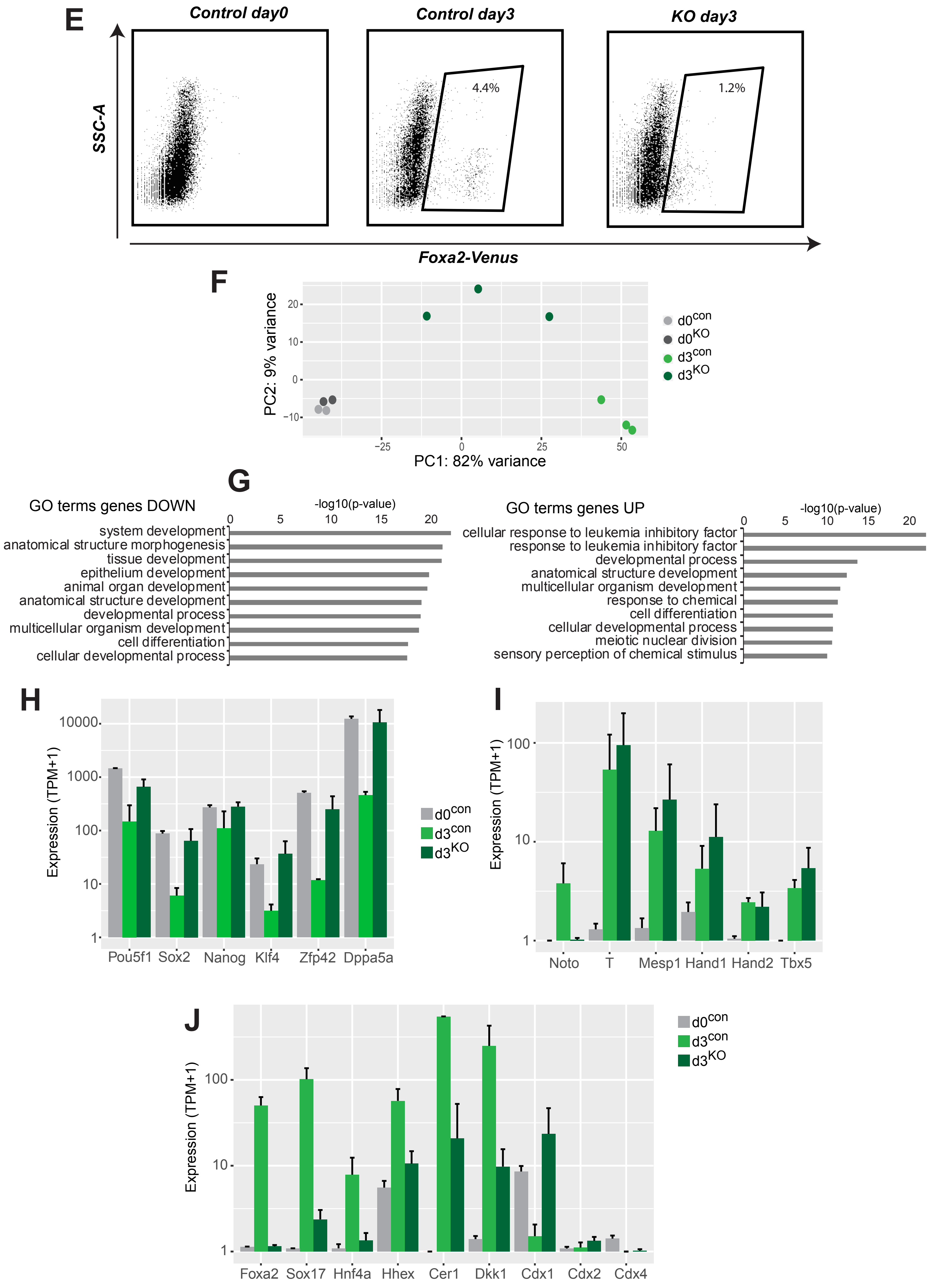

Supplement related to Figure 4

A

Top motifs at Transient Foxa2 peaks

Rank	Homer Known Motif	Name	p-val	% of targets
1	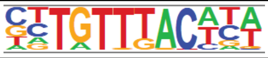	Foxa2	1e-1655	70.86

Top motifs at Stable Foxa2 peaks

Rank	Homer Known Motif	Name	p-val	% of targets
1	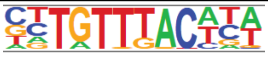	Foxa2	1e-3037	82.70

Top motifs at Late Foxa2 peaks

Rank	Homer Known Motif	Name	p-val	% of targets
1	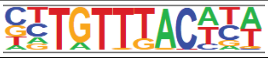	Foxa2	1e-1996	74.87

B

GO Biological Process at Transient Foxa2 peaks

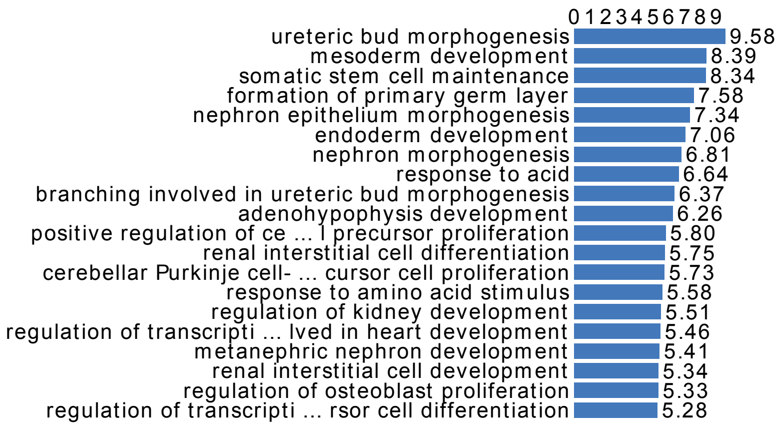

GO Biological Process at Stable Foxa2 peaks

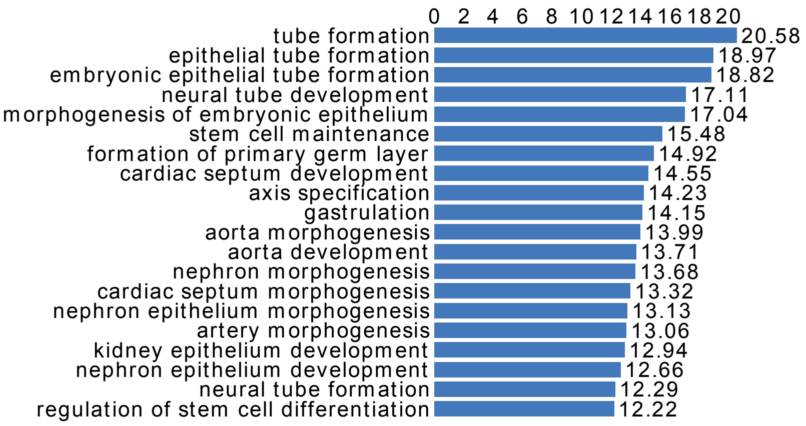

GO Biological Process at Late Foxa2 Peaks

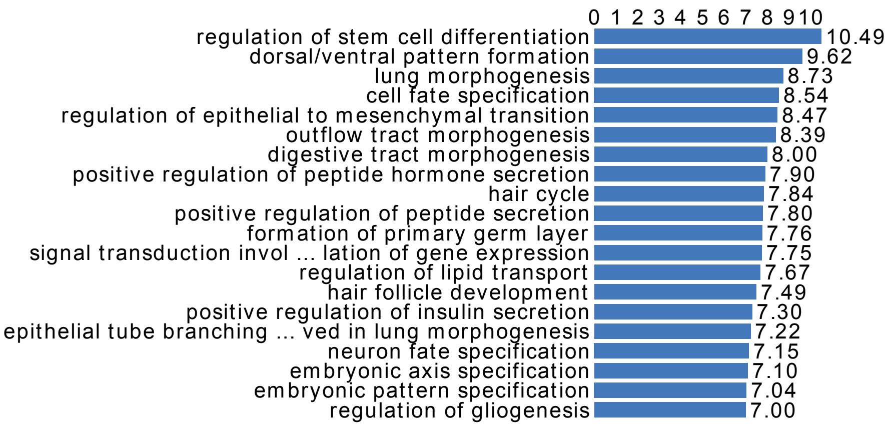

Supplement related to Figure 4

C

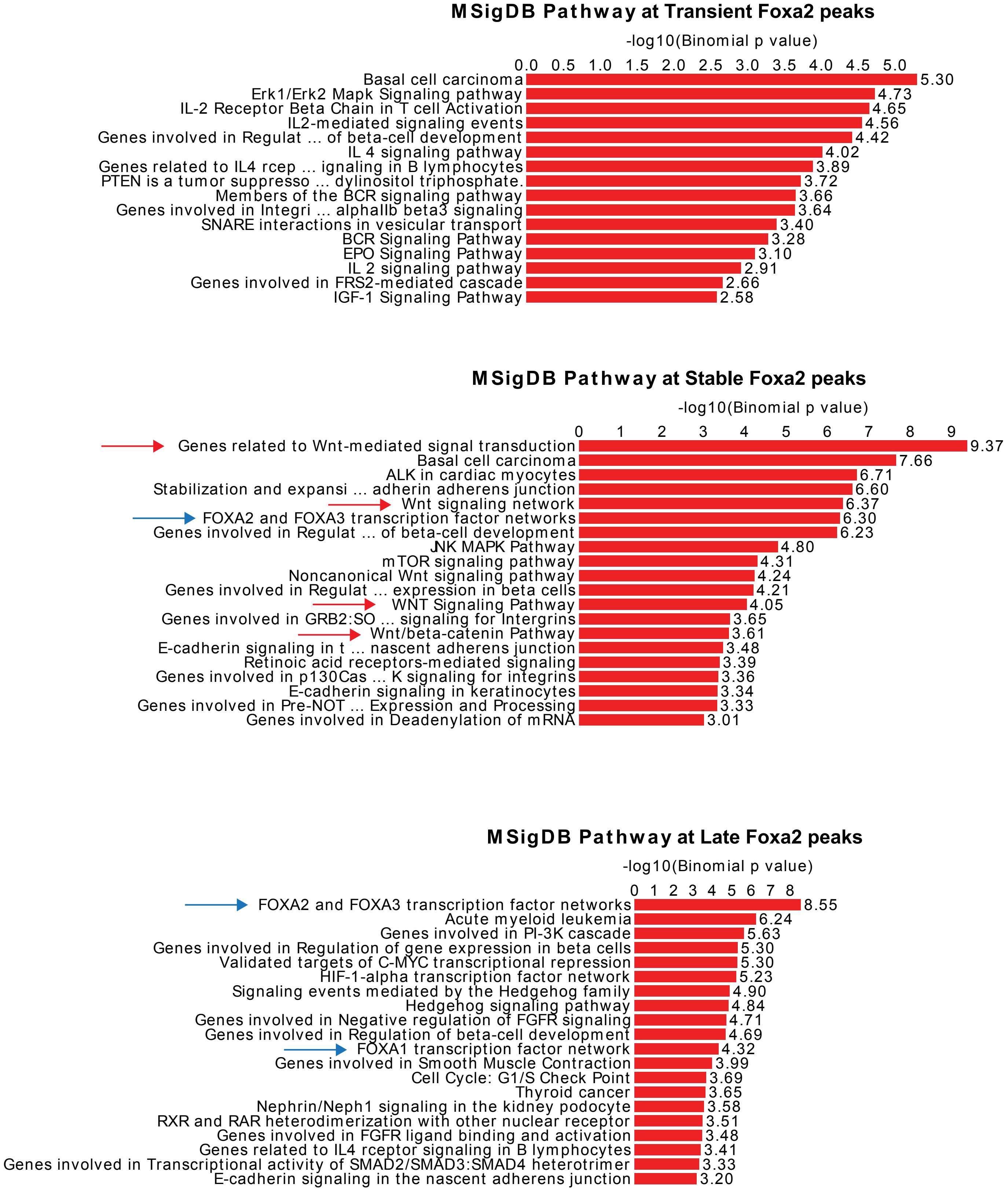

Supplement related to Figure 4

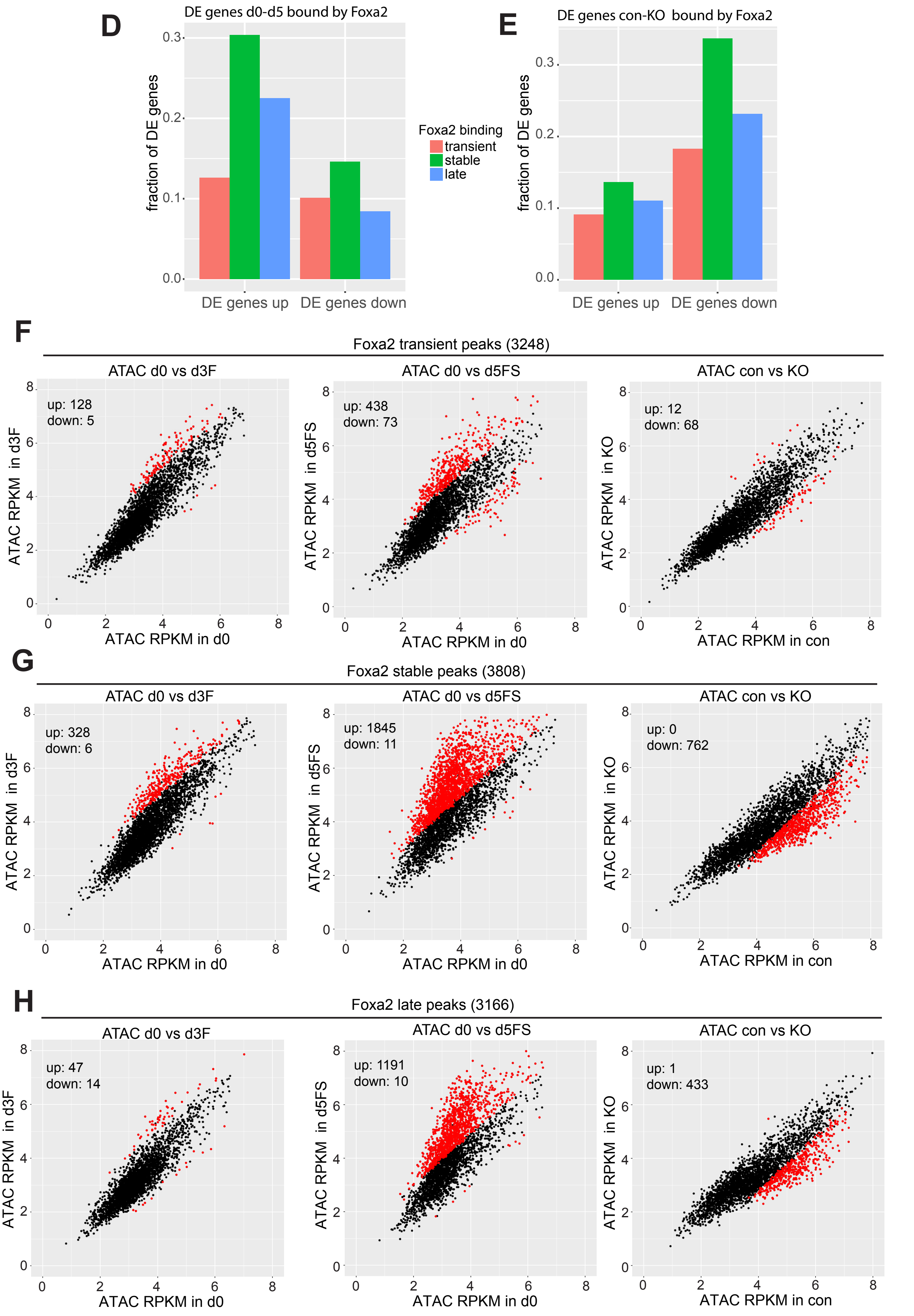

Supplement related to Figure 4

I

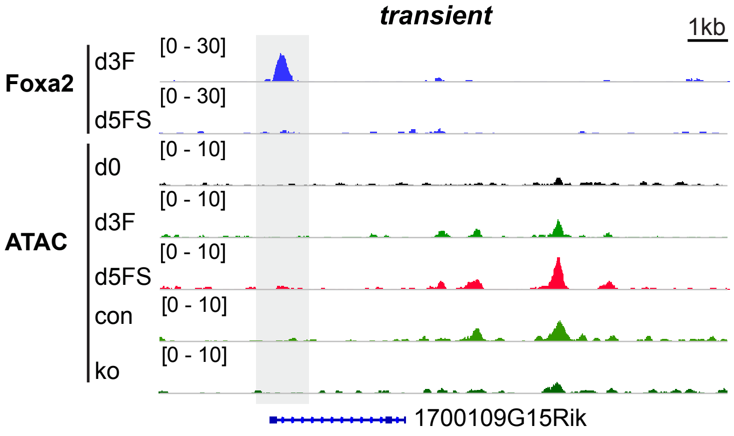

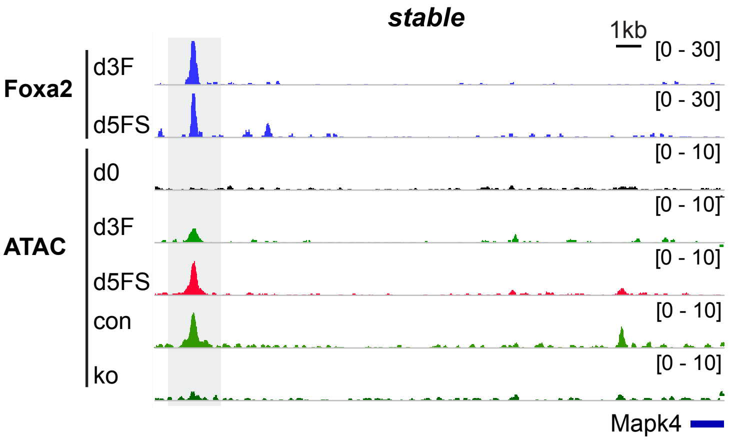

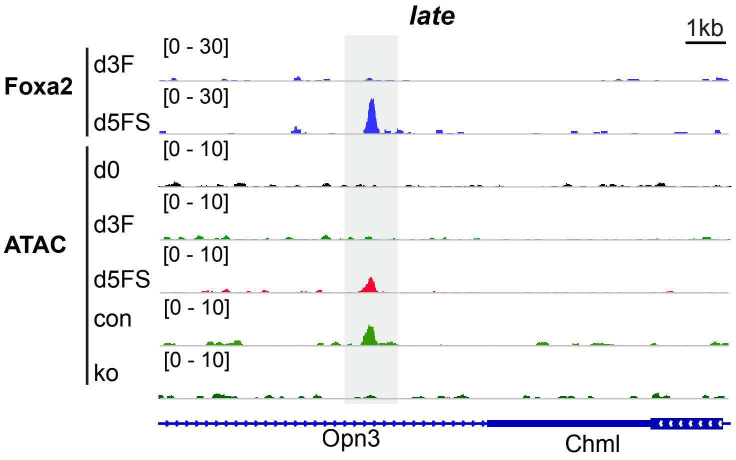

Supplement related to Figure 4

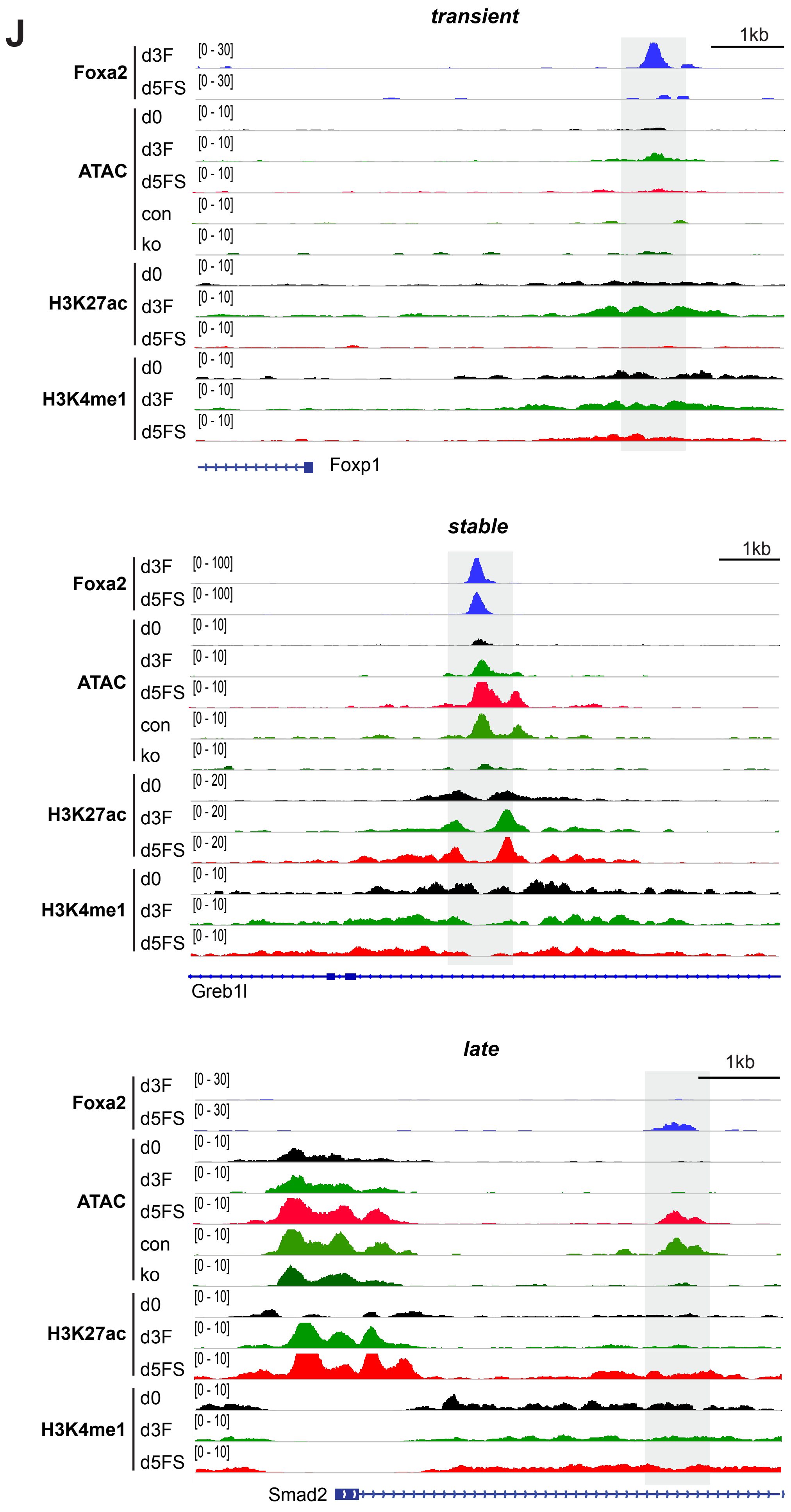

Supplement related to Figure 5

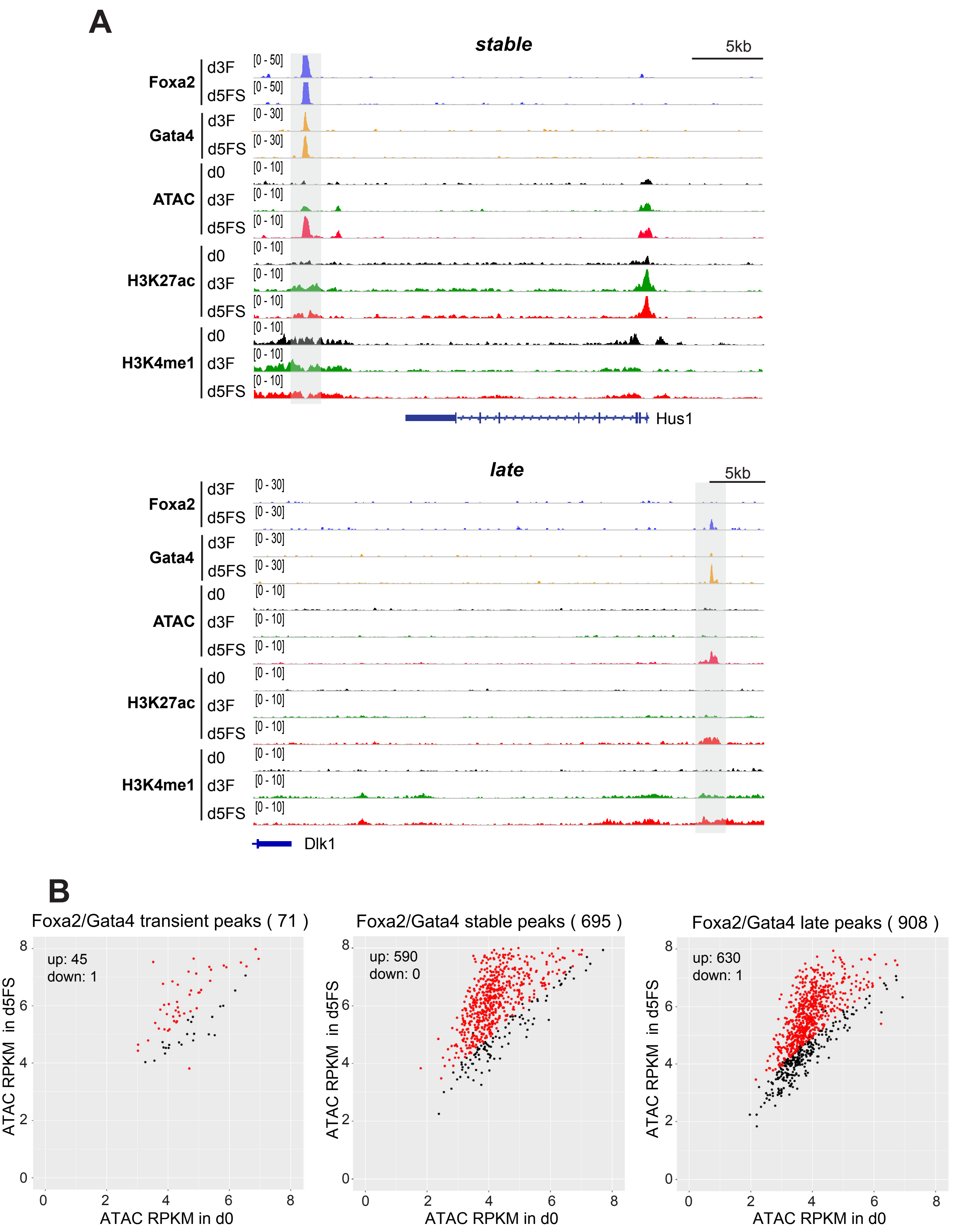

Supplement related to Figure 6

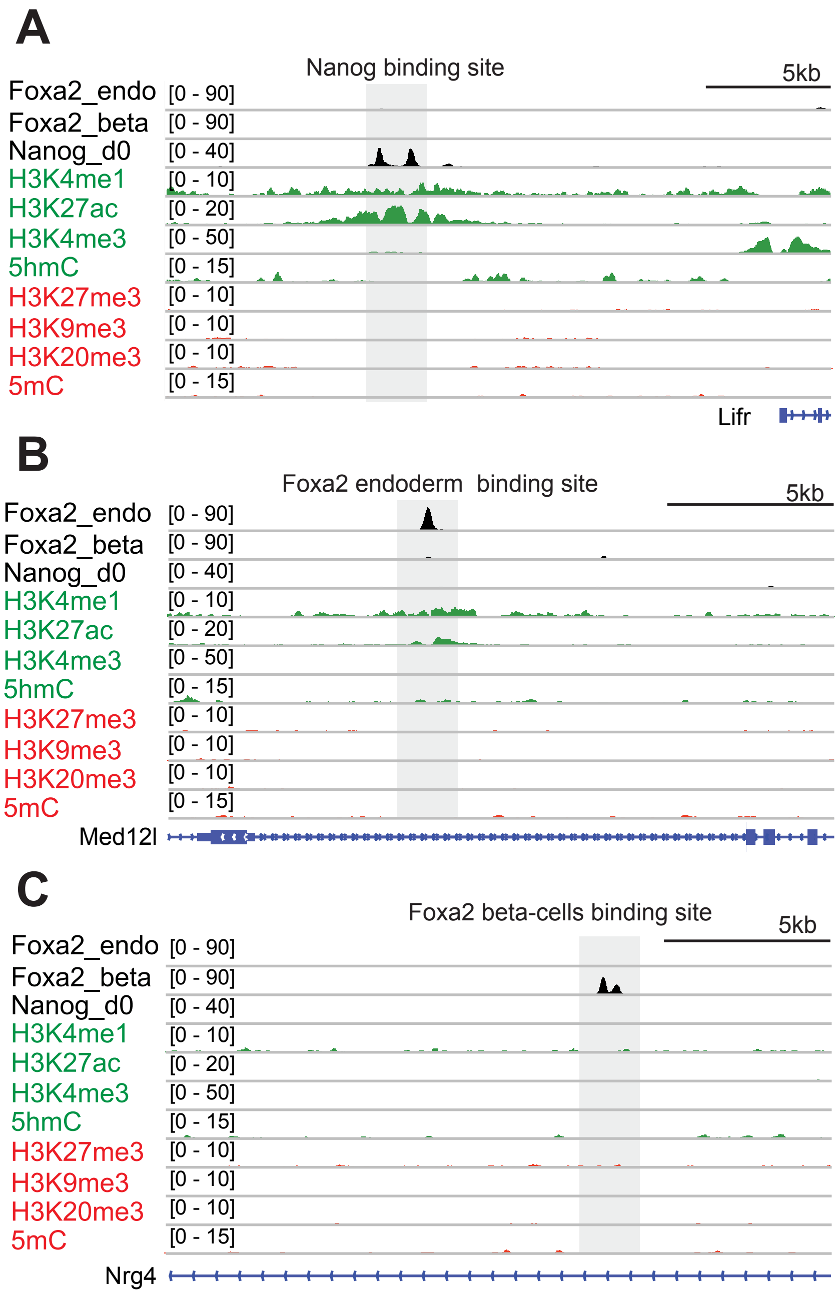

Supplement related to Figure 6

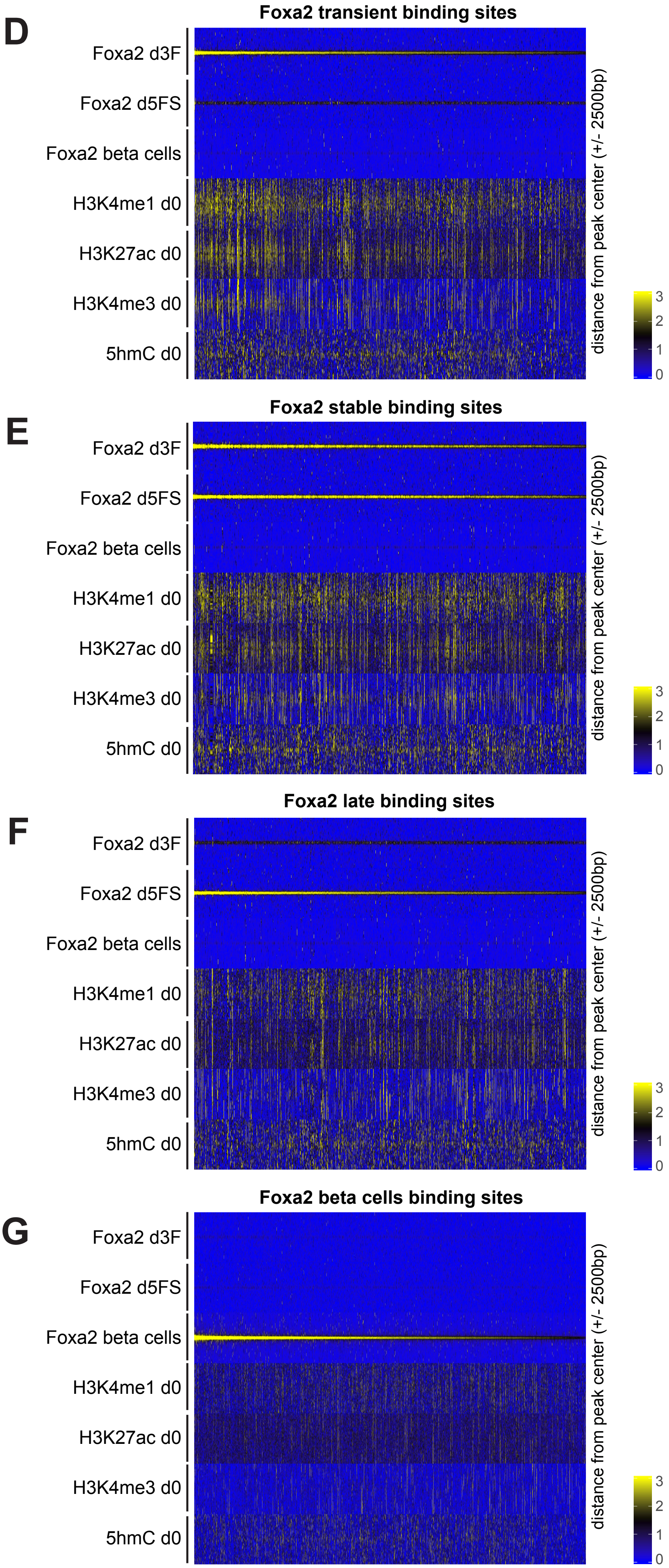

Supplement related to Figure 6

H

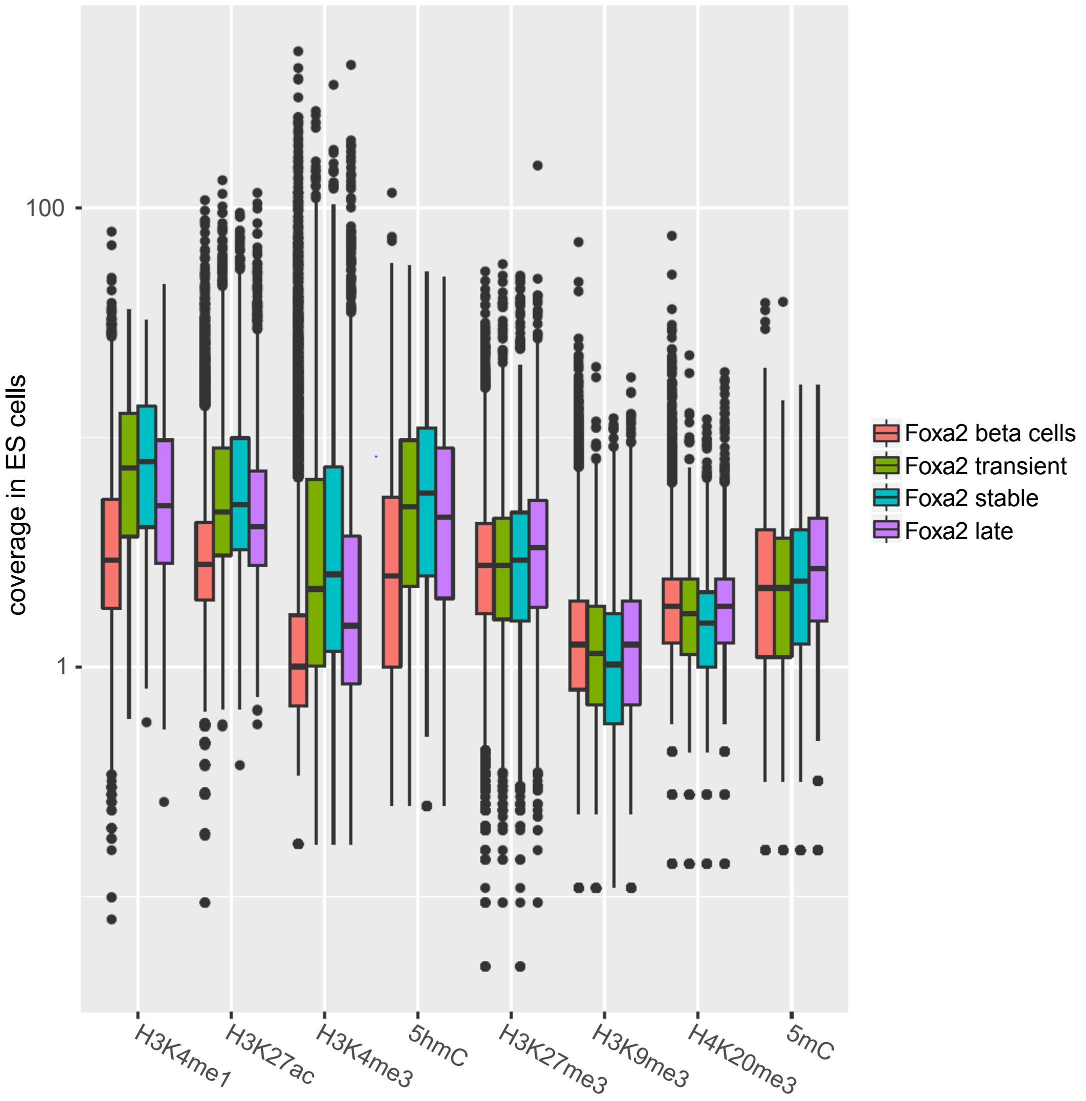

I

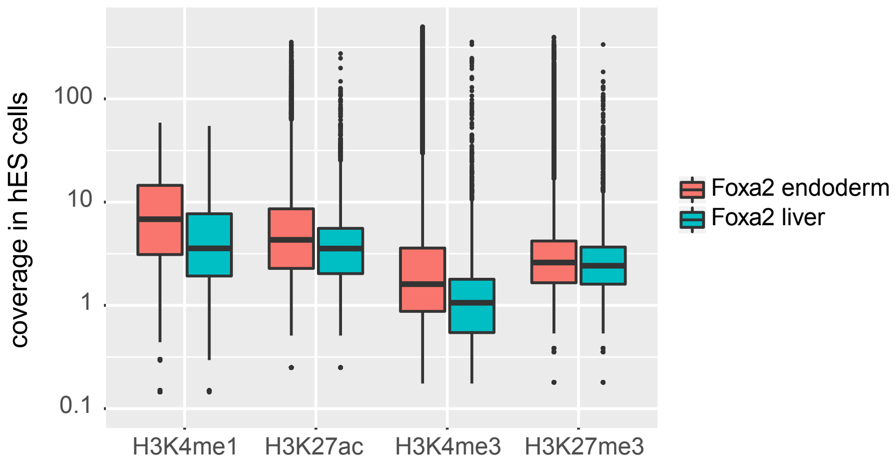

Supplement related to Figure7

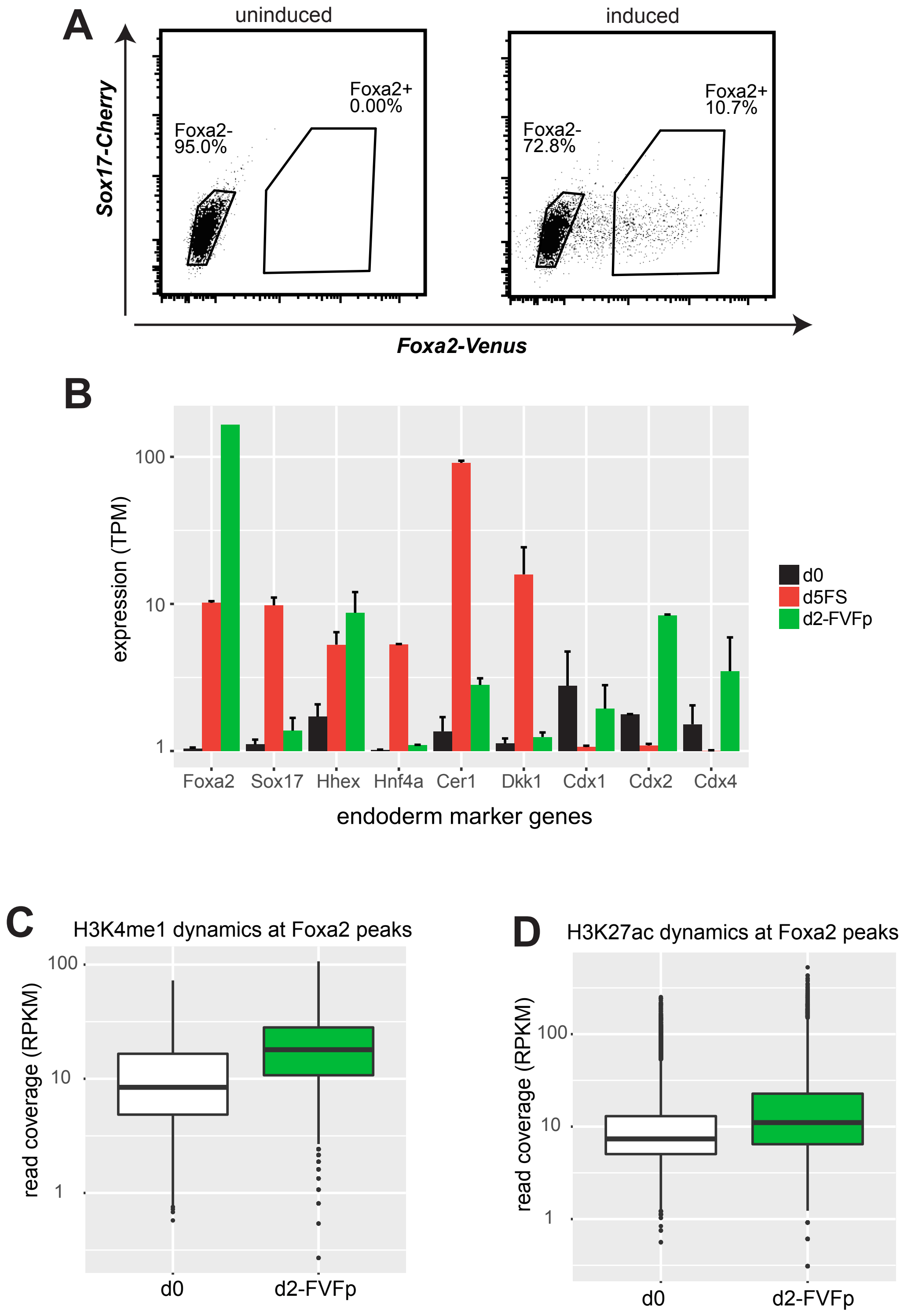

Supplement related to Figure7

E

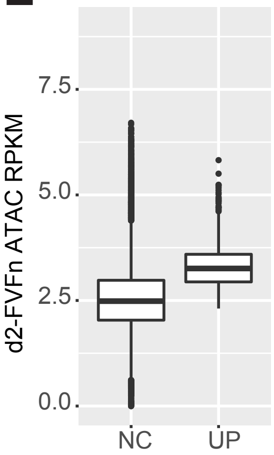

F

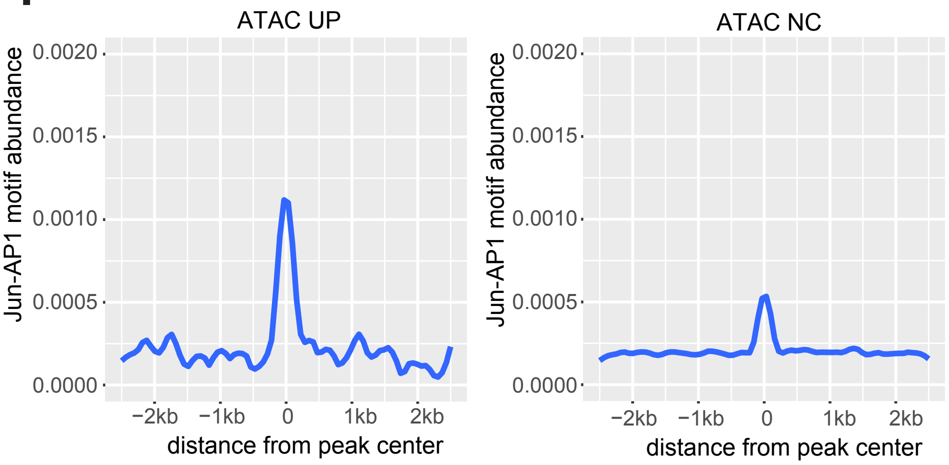

G

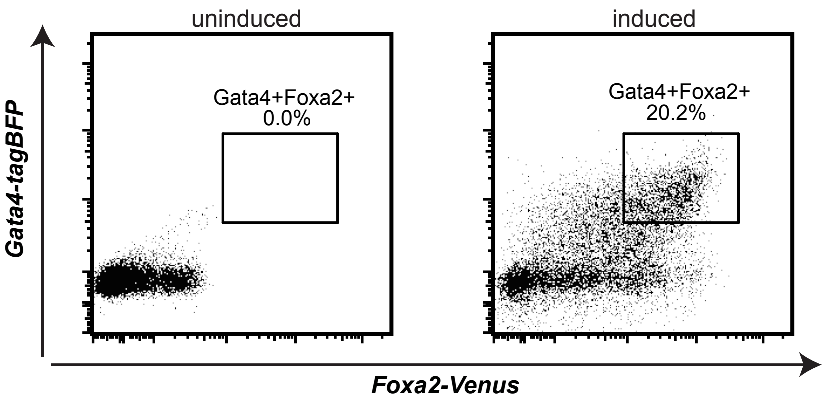

REFERENCES

1. Nagai, T., Ibata, K., Park, E.S., Kubota, M., Mikoshiba, K. and Miyawaki, A. (2002) A variant of yellow fluorescent protein with fast and efficient maturation for cell-biological applications. *Nat Biotechnol*, **20**, 87-90.
2. Burtscher, I., Barkey, W. and Lickert, H. (2013) Foxa2-venus fusion reporter mouse line allows live-cell analysis of endoderm-derived organ formation. *Genesis*, **51**, 596-604.
3. Hitz, C., Wurst, W. and Kuhn, R. (2007) Conditional brain-specific knockdown of MAPK using Cre/loxP regulated RNA interference. *Nucleic Acids Res*, **35**, e90.
4. Sadic, D., Schmidt, K., Groh, S., Kondofersky, I., Ellwart, J., Fuchs, C., Theis, F.J. and Schotta, G. (2015) Atrx promotes heterochromatin formation at retrotransposons. *EMBO Rep*, **16**, 836-850.
5. Gibson, D.G., Young, L., Chuang, R.Y., Venter, J.C., Hutchison, C.A., 3rd and Smith, H.O. (2009) Enzymatic assembly of DNA molecules up to several hundred kilobases. *Nat Methods*, **6**, 343-345.
6. Dobin, A., Davis, C.A., Schlesinger, F., Drenkow, J., Zaleski, C., Jha, S., Batut, P., Chaisson, M. and Gingeras, T.R. (2013) STAR: ultrafast universal RNA-seq aligner. *Bioinformatics*, **29**, 15-21.
7. Love, M.I., Huber, W. and Anders, S. (2014) Moderated estimation of fold change and dispersion for RNA-seq data with DESeq2. *Genome Biol*, **15**, 550.
8. Langmead, B. (2010) Aligning short sequencing reads with Bowtie. *Curr Protoc Bioinformatics*, **Chapter 11**, Unit 11 17.
9. Heinz, S., Benner, C., Spann, N., Bertolino, E., Lin, Y.C., Laslo, P., Cheng, J.X., Murre, C., Singh, H. and Glass, C.K. (2010) Simple combinations of lineage-determining transcription factors prime cis-regulatory elements required for macrophage and B cell identities. *Mol Cell*, **38**, 576-589.
10. Rackham, O.J., Firas, J., Fang, H., Oates, M.E., Holmes, M.L., Knaupp, A.S., Consortium, F., Suzuki, H., Nefzger, C.M., Daub, C.O. *et al.* (2016) A predictive computational framework for direct reprogramming between human cell types. *Nat Genet*, **48**, 331-335.
11. Szklarczyk, D., Morris, J.H., Cook, H., Kuhn, M., Wyder, S., Simonovic, M., Santos, A., Doncheva, N.T., Roth, A., Bork, P. *et al.* (2017) The STRING database in 2017: quality-controlled protein-protein association networks, made broadly accessible. *Nucleic Acids Res*, **45**, D362-D368.
12. Shannon, P., Markiel, A., Ozier, O., Baliga, N.S., Wang, J.T., Ramage, D., Amin, N., Schwikowski, B. and Ideker, T. (2003) Cytoscape: a software environment for integrated models of biomolecular interaction networks. *Genome Res*, **13**, 2498-2504.
13. McLean, C.Y., Bristor, D., Hiller, M., Clarke, S.L., Schaar, B.T., Lowe, C.B., Wenger, A.M. and Bejerano, G. (2010) GREAT improves functional interpretation of cis-regulatory regions. *Nat Biotechnol*, **28**, 495-501.
14. Mi, H., Huang, X., Muruganujan, A., Tang, H., Mills, C., Kang, D. and Thomas, P.D. (2017) PANTHER version 11: expanded annotation data from Gene Ontology and Reactome pathways, and data analysis tool enhancements. *Nucleic Acids Res*, **45**, D183-D189.
